# Modelling the impacts of climate change on thermal habitat suitability for shallow-water marine fish at a global scale

**DOI:** 10.1371/journal.pone.0258184

**Published:** 2021-10-04

**Authors:** Edward Lavender, Clive J. Fox, Michael T. Burrows

**Affiliations:** The Scottish Association for Marine Science, Scottish Marine Institute, Dunstaffnage, Oban, Argyll, Scotland; Instituto Federal de Educacao Ciencia e Tecnologia Goiano - Campus Urutai, BRAZIL

## Abstract

Understanding and predicting the response of marine communities to climate change at large spatial scales, and distilling this information for policymakers, are prerequisites for ecosystem-based management. Changes in thermal habitat suitability across species’ distributions are especially concerning because of their implications for abundance, affecting species’ conservation, trophic interactions and fisheries. However, most predictive studies of the effects of climate change have tended to be sub-global in scale and focused on shifts in species’ range edges or commercially exploited species. Here, we develop a widely applicable methodology based on climate response curves to predict global-scale changes in thermal habitat suitability. We apply the approach across the distributions of 2,293 shallow-water fish species under Representative Concentration Pathways 4.5 and 8.5 by 2050–2100. We find a clear pattern of predicted declines in thermal habitat suitability in the tropics versus general increases at higher latitudes. The Indo-Pacific, the Caribbean and western Africa emerge as the areas of most concern, where high species richness and the strongest declines in thermal habitat suitability coincide. This reflects a pattern of consistently narrow thermal ranges, with most species in these regions already exposed to temperatures above inferred thermal optima. In contrast, in temperate regions, such as northern Europe, where most species live below thermal optima and thermal ranges are wider, positive changes in thermal habitat suitability suggest that these areas are likely to emerge as the greatest beneficiaries of climate change, despite strong predicted temperature increases.

## Introduction

Climate change is increasingly impacting marine ecosystems [1]. The main physical impacts are temperature change, oxygen depletion and ocean acidification—the so-called ‘deadly trio’ [2]. These changes affect the physiology and performance of marine organisms [3]. For example, rising temperatures increase oxygen demand while reducing oxygen supply, constraining aerobic performance [4]. These physiological impacts can have major consequences for population abundance, community diversity and ecosystem structure and function [5].

From a conservation perspective, changes in abundance are particularly concerning. Substantial reductions in abundance increase population vulnerability to intrinsic and extrinsic stressors, including anthropogenic pressures, such as fishing, alongside demographic and environmental stochasticity [6–9]. Thus, reduced abundance is linked to increased risks of stock collapse and reduced recovery potential [6–9]. At the same time, abundance changes may affect trophic interactions, ecosystem functioning and the delivery of ecosystem services, such as fisheries yields [6, 10, 11].

Some communities are especially vulnerable to these changes as a result of their exposure, sensitivity and limited adaptive capacity [12]. Many tropical communities stand out as areas of concern, with diverse marine communities that are already under pressure [13–16]. With climate change, these pressures may continue to worsen, particularly in shallow, inshore areas in which temperature changes are likely to be realised most quickly [17]. At the same time, many tropical communities are dependent on healthy fish populations as sources of protein, nutrients and income, with limited adaptive capacity [12, 18–20].

If ecosystems are to be exploited and managed sustainably, we need to understand the developing threats and drivers of change in marine communities, and which areas are likely to be most strongly affected [21–23]. Large-scale predictions of the impacts of climate change underpin regional food security, socioeconomic and vulnerability assessments [12, 24, 25]. Collectively, these assessments form the basis for addressing conservation and food security targets and highlighting areas in which adaptive capacity needs to be strengthened [26–28].

At large spatial scales, temperature appears to be one of the most important drivers of abundance change in marine communities through its influence on thermal habitat suitability [29]. In the North Sea, scientific trawl data suggest that species with southerly biogeographic affinities, such red mullet (*Mullus barbatus*), anchovy (*Engraulis encrasicolus*) and pilchard (*Sardina pilchardus*), are increasing in abundance, whilst boreal species are declining [30]. Across the European continental shelf, 72% of the 50 most abundant demersal fish species show significant relationships between abundance and temperature [31]. Likewise, on the North East United States continental shelf, shifts in the centre of biomass of many fish stocks are associated with large-scale temperature increases and changes in circulation [32]. At a global scale, increasing tropicalisation of commercial catches has been documented as the proportion of warm-water species in catches has increased [33].

Yet while evidence for temperature-driven change in marine communities is accumulating across the globe, predictive studies of future changes have tended to be sub-global in scale [30, 34, 35] or predominantly focussed on shifts in species’ ranges [36–38] or commercially exploited species [39, 40]. Fewer studies have considered the consequences of temperature change across species’ distributions at a global scale, and these have also tended to focus on commercial species [23, 28, 29, 39–43]. Consequently, there is a need to continue to develop global-scale modelling approaches, particularly those that can be applied to non-commercial species in the tropics [12, 29].

**Fig 1:**
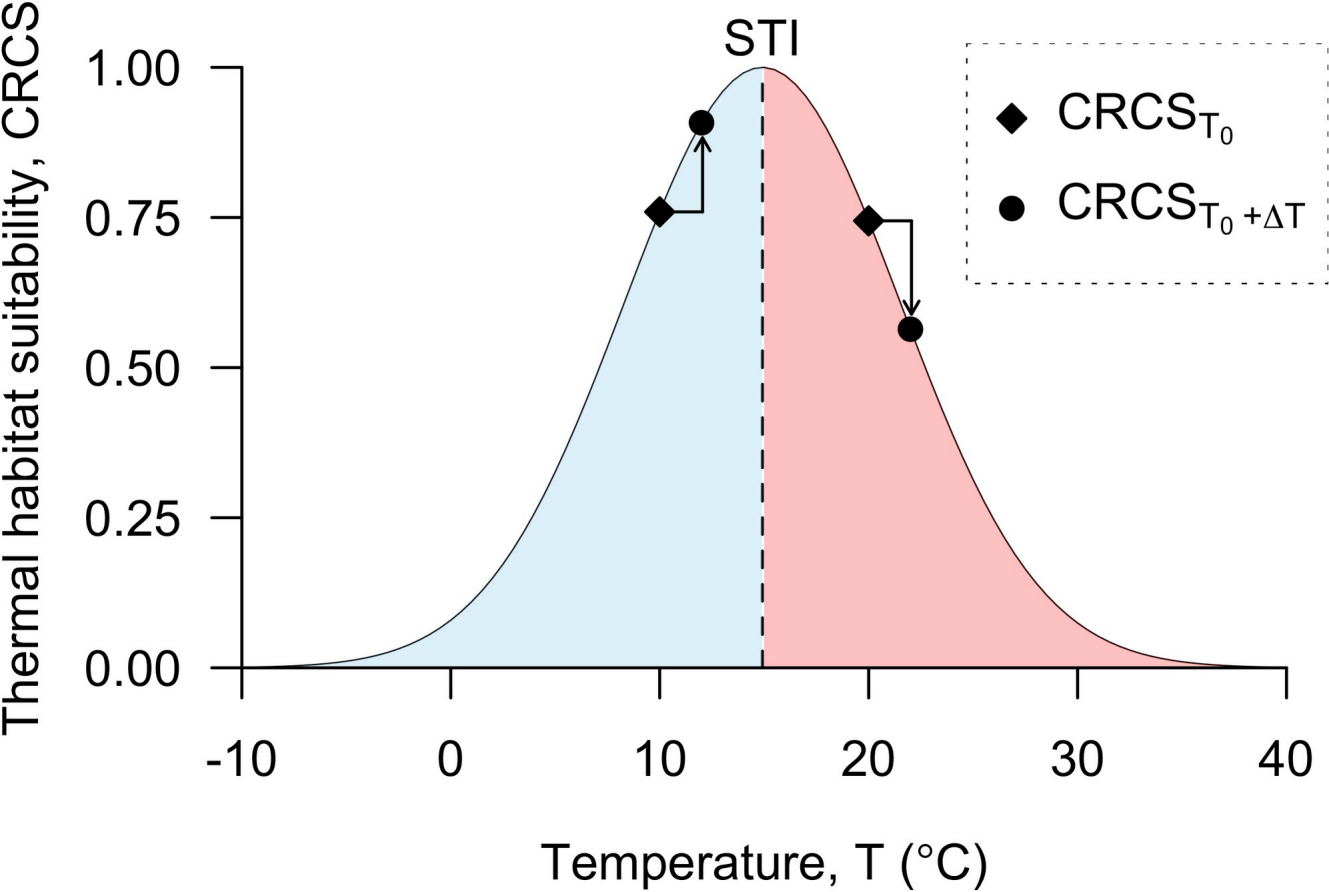
A Gaussian climate response curve. This curve is based on the 10^th^, 50^th^ and 90^th^ quantiles in the baseline sea surface temperatures (SSTs) [50] occupied by Atlantic sturgeon (*Acipenser oxyrinchus*) [51]. The estimated 50^th^ quantile (the species’ thermal index, STI) is shown by the vertical dashed line and labelled. Below the STI, the ‘cold’ half of the distribution is shown in blue; an increase in temperature for populations occupying these temperatures (for example, from 10 to 12°C, shown) is assumed to lead to an increase in the CRCS. In contrast, above the STI, the ‘warm’ half of the distribution is shown in red; an increase in temperature for populations occupying these temperatures (for example, from 20 to 22°C, shown) is assumed to lead to a decline in the CRCS. The diamond-shaped points mark the predicted CRCS for two hypothetical populations, each occupying an example starting temperature *T*_*0*_; the circular points mark the CRCS of those populations after an assumed 2°C rise in temperature (Δ*T*) following climate change, at temperature *T*_*0*_ *+ ΔT*. Arrows show the assumed direction of change in the CRCS for these two hypothetical populations in the cold and warm halves of the distribution.

One possible approach is based on species’ thermal affinities. These can be inferred from the quantiles of observed variation in temperature across species’ distributions [44]. Thermal affinities can be used to parameterise climate response curves, which relate temperature to an index of thermal habitat suitability, here termed the climate response curve suitability (CRCS) [45, 46]. Under a Gaussian climate response curve, a species is assumed have an optimum temperature, which can be estimated as the median temperature across the species’ distribution and termed the species’ thermal index (STI), with the CRCS declining symmetrically with deviations above or below this optimum [47–49] (Fig 1). Under this model, future temperature increases are assumed to lead to an increase in the CRCS for populations below the STI and a decline in the CRCS for populations above the STI (Fig 1).

The central assumption of the Gaussian climate response curve is that the relationship between temperature and the CRCS follows a Gaussian distribution, with the CRCS maximised at the STI [47, 52]. This relationship is reflected in the Abundance-Centre Hypothesis [3, 53]. In the context of climate change predictions, this hypothesis is akin to a space-for-time substitution in which the drivers of species’ distributions are assumed to drive temporal change too [54, 55]. This hypothesis has been controversial, but recent work is generally supportive [49, 55–58].

Building on this assumption, Gaussian climate response curves offer a means to generate predictions about broad-scale changes in the CRCS of a large number of species, including poorly studied, non-commercial species, under future temperature change. A particular strength of the approach is that the relative importance of different drivers of change is tractable. Specifically, spatial variation in species’ sensitivity to temperature change depends on spatial variation in three variables: (1) the magnitude of temperature change; (2) species’ thermal ranges (STRs); and (3) the disparity between optimal and current temperatures (species’ thermal biases, STBs [56]). Increased temperatures are expected to lead to greater changes in the CRCS, but these depend on STRs and the direction and magnitude of STBs. For example, for the same magnitude of temperature change and STRs, species living below their optimum temperature are less likely to experience declines in CRCS under warming than species living above their optimum temperature [56]. The relative contribution of these drivers of species’ sensitivity to climate change across space is an ongoing area of research to which this approach can contribute [56].

The wider utility of this approach depends on the hypothesis that changes in temperature, through their influence on thermal habitat suitability, broadly correspond with changes in important biogeographic patterns, especially abundance, at large spatial scales. There are many other direct and indirect consequences of climate change that can affect abundance, including altered weather patterns, ocean acidification, hypoxia and modified species interactions, such as competition and prey availability [59–63]. For example, interannual monsoon wind variability is a key driver of small pelagic fisheries yields in East Africa through changes in stratification and primary production [64]. Likewise, within inshore environments, increases in precipitation intensity may increase terrestrial run-off, nutrient input and turbidity, affecting fish populations [65]. Yet while other climatic drivers of change are important, these often covary with temperature [4]. Furthermore, while the effects of climate change may be modulated by species interactions, their effects tend to be localised [59–61]. There is compelling evidence that temperature and thermal habitat suitability are dominant drivers of species’ distributions and relative abundance over broad spatiotemporal scales [29, 31, 33, 58]. For example, an analysis of 1,790 marine species’ distributions suggested temperature is the limiting factor constraining species’ ranges [66]. Accordingly, climate velocities can predict the direction and rate of range shifts for many marine species [67, 68]. Likewise, temperature change and thermal affinities successfully predict observed trends in relative abundance and community turnover from regional to ocean scales, notwithstanding other drivers of change [58, 69, 70]. Taken together, these studies suggest that, on average, across many species, the influence of temperature on thermal habitat suitability is likely to drive corresponding changes in relative abundance, although other influences may mask this effect at the level of individual species or local spatial scales [55]. This strongly supports the utility of the climate response curves for modelling the impacts of climate change in marine ecosystems.

Here, we develop the Gaussian climate response curve approach to provide predictions for change in the CRCS of shallow-water marine fish species under two climate experiments over a spatiotemporal scale relevant to policymakers. The objectives are as follows:

1. Predict the change in the CRCS under mid-century and late-century timescales for Representative Concentration Pathways (RCPs) 4.5 and 8.5 [65] for coastal, shallow-water marine fish at a global scale.
2. Synthesise these predictions across the coastal areas of Exclusive Economic Zones (EEZs) to identify those regions most at risk and those which may benefit from climate change.
3. Compare the relative importance of temperature change, STRs and STBs as drivers of these patterns.

## Materials and methods

### Study species

We restricted our study to coastal, shallow-water fish species. These species are particularly exposed to anthropogenic impacts [15] and represent one of the most accessible exploited resources and an important source of income in many of the regions that are most vulnerable to climate change [12, 20]. Additionally, the ambient thermal experience of shallow-water fish is likely to reflect closely sea surface temperatures (SSTs), while that of vertically mobile species that move between depth layers may be affected more strongly by temperature gradients throughout the water column (e.g., [71, 72]).

To define a set of species to model, we examined depth preferences and species’ distributions for an initial list of 17,343 marine species. Species’ depth limits were queried from FishBase, using the rfishbase package in R, version 4.0.2 [73, 74]. We defined shallow-water species conservatively as those for which the deepest reported depth was less than 50 m. While thermal stratification can occur in water shallower than 50 m, the median depth for the vast majority of these species was considerably shallower than this. In addition, alongside SST, we evaluated the utility of sea bottom temperatures (SBTs) for the characterisation of species’ thermal affinities (see below).

Aquamaps species’ distributions were used to describe the distribution of each species [44, 51]. This is a widely used, global-scale species’ distribution modelling (SDM) framework. The approach predicts the relative probability of species’ occurrences across a 0.5 x 0.5° global grid, based on habitat suitability envelopes and, for some well-studied species, expert knowledge. We obtained Aquamaps SDM predictions for modelled species using the aquamapsdata R package [51]. We only considered species for which SDMs were based on occurrence observations in more than 10 unique cells, thus excluding species with highly uncertain SDMs [51]. SDM predictions were masked by the world coastline, using public domain data from Natural Earth, and inspected in relation to occurrence data obtained from the Ocean Biodiversity Information System and the Global Biodiversity Information Facility via the robis and rgbif packages [75–77]. Species with obvious discontinuities along their range edges, which result in some circumstances from the way distributions are constrained to Major Fishing Areas by Aquamaps, were excluded. For the final list of species, we obtained data from Fishbase via rfishbase to examine their importance to people.

### Temperature projections

Baseline and future temperature projections were obtained from the National Oceanographic and Atmospheric Administration (NOAA) Climate Change Web Portal for Coupled Model Intercomparison Project Phase 5 (CMIP5) models [50]. This service provides ensemble-average projections from CMIP5 models, re-scaled to one-degree resolution, for a baseline period (1956–2005), a mid-century period (2006–2055) and a late-century period (2050–2099). Ensemble projections were used for both baseline and future temperatures to ensure that temperatures for all time windows were subject to the same biases and to minimise the sensitivity of our predictions to inter-model and temporal variability [78]. There are other temperature metrics, such as seasonal temperatures or thermal extremes, that may be better specific predictors of thermal habitat suitability, but these metrics tend to covary with the average temperature and the latter is more widely applicable [79–81].

We obtained baseline SSTs from this service alongside mid- and late-century projections for two climate experiments: RCP 4.5 (an intermediate greenhouse gas concentration scenario in which emissions continue to rise until approximately 2040 before declining) and 8.5 (a ‘business-as-usual’ greenhouse gas concentration scenario in which emissions continue to rise throughout this century). We also obtained baseline and future SBT projections to evaluate their utility for characterising thermal affinities and prediction. For consistency with SDMs, projections were re-sampled to 0.5° resolution using bilinear interpolation and masked by the world coastline.

### Species’ thermal affinities

Weighted and unweighted thermal affinities were estimated across each species’ distribution using (a) SST and (b) SBT baseline temperatures. Weighted thermal affinities were estimated as the 10^th^, 50^th^ and 90^th^ quantiles in baseline temperatures (denoted T_10_, STI and T_90_) across each species’ distribution, using predicted occupancy probabilities as weights. As in other studies [58], we used these quantiles because they are less sensitive to the edges of species’ distributions, which tend to be more uncertain. Unweighted thermal affinities were estimated by discretising probabilistic species’ distributions into areas of ‘presence’, where the relative probability of occupancy was at least 0.5, and areas of ‘absence’, and then estimating the quantiles of temperature variation across areas of ‘presence’.

In subsequent analysis, as in other studies [82, 83], we used unweighted thermal affinities and predicted changes across discretised distributions based on the 50% probability threshold. Binary range maps can correspond more closely with areas of known occurrence [84] and were computationally tractable, whereas predicting changes across all areas with a non-zero probability of species occupancy and weighting predictions by the probability of occupancy was computationally infeasible.

### Species’ sensitivity

We evaluated spatial patterns in species’ sensitivity to projected temperature changes by mapping spatial variation in projected temperature changes, STRs and STBs across a global grid. STRs were defined as the difference between the upper and lower thermal affinities (*T*_*90*_ *–T*_*10*_) [58]. STBs were defined as the difference between baseline temperatures and STIs [56].

### Model predictions

For each species, unweighted thermal affinities were used to parameterise a Gaussian climate response curve relating the CRCS to SST or SBT, with the mean equal to the STI and the standard deviation defined as:

$$\sigma =\frac{T_{90}-T_{10}}{2.563}$$ (1)

where *T*_*90*_ and *T*_*10*_ are the 90^th^ and 10^th^ quantiles of variation in baseline temperatures across the discretised species’ distribution. For both SST and SBT, for both baseline temperatures and the two RCP experiments, we used these values to predict the CRCS (scaled between 0 and 1) of each species (*s*) in each grid cell (*i*, *j*) based on the temperature (*T*) in that cell, according to the equation:

$$CRCS_{i,j,s}=e^{-\left[\frac{{\left(T_{i,j}-STI_{S}\right)}^{2}}{2\sigma_{S}^{2}}\right]}$$ (2)

where *σ*_*s*_ is the standard deviation for a given species [Eq (1)]; and *STI*_*s*_ is the Species’ Thermal Index for species *s*.

We synthesised predicted changes in the CRCS between baseline and projected temperatures across species in each grid cell using two metrics: (1) the mean change in the CRCS across all species (denoted E(ΔCRCS_*i*,*j*_)) and (2) the proportion of species predicted to experience declines in the CRCS (denoted Pr(ΔCRCS_*i*,*j*_ < 0)). We also synthesised overall trends across the coastal areas of all EEZs that contained more than five modelled species by (1) averaging the mean change in the CRCS in each grid cell across all grid cells containing modelled species in each EEZ (denoted E(ΔCRCS_*EEZ*_)) and (2) calculating the total proportion of species predicted to experience declines in each EEZ (denoted Pr(ΔCRCS_*EEZ*_ < 0)), using EEZ boundary data from the Maritime Boundaries Geodatabase [85]. In both analyses, predictions were driven by modelled shallow-water species and reflect changes in coastal areas.

### Comparison of climate experiments

Predicted changes in the CRCS for modelled species were examined visually across the globe and across EEZs to identify differences between the climate experiments and mid- and late-century timescales. For EEZs with more than modelled five species, we quantified the extent to which predicted changes in the CRCS under RCP 8.5 were greater than under RCP 4.5 for the late-century time window by modelling the mean change in the CRCS in each EEZ coastal zone (E(ΔCRCS_*EEZ*_)) under RCP 8.5 as a function of the change under RCP 4.5 in a robust linear regression framework, as implemented by the MASS package [86], with the number of species modelled in each EEZ used as weights. We also compared how the proportion of species projected to experience declines in each EEZ differed between experiments using density distributions.

## Results

### Study species

2,293 species passed data processing filters, including 2,271 actinopterygians and 22 elasmobranchs (S1 Table). Among modelled species, the average median depth (calculated from species’ shallow and deep limits) is 13.01 m and the 95^th^ quantile in species’ shallow and deep depth limits is 12 and 44 m respectively (S1 Fig). Selected species are restricted to inshore regions along coastlines and islands, with species richness concentrated in the Indo-Pacific (S2 Fig). Reef-associated species (n = 1,165) predominate, but species with other lifestyles, including demersal and pelagic species, are included. Among the 36.50% of species for which information on their importance to people is available on FishBase, the ‘highly commercial’ (1.19%), ‘commercial’ (23.54%) and ‘minor commercial’ (39.18%) categories account for approximately two thirds, with a further 6.18% of species flagged as ‘subsistence’. The absence of data on the importance to people of the remaining 63.50% of modelled species suggests that most are probably not commercially targeted, but their importance in other settings remains uncertain.

### Temperature projections

Baseline SSTs in coastal regions range from -1.96°C at the poles to 29.80°C in the tropics (S3A Fig). Similarly, SBTs vary between -1.88–30.40°C, but are less strongly structured by latitude (S3B Fig). In particular, while SBTs are highest in shallow parts of the Indo-Pacific, especially in Australasia and Sundaland, in deeper equatorial areas, including around Wallacea and East Melanesia, SBTs are considerably cooler. Warmer SBTs are more commonly found along the Tropics of Cancer and Capricorn, particularly in the Caribbean, East Asia and eastern Australia.

SST and SBT within EEZs are projected to warm by up to 5.05°C over the 21^st^ century (S4 and S5 Figs). Under RCP 4.5, SST is projected to warm by an average 0.69–1.26°C over mid- to late-century timescales respectively (S4 Fig). Under RCP 8.5, projected changes are more extreme, with mean warming of 0.79–2.08°C. In both climate experiments, mid-century warming is strongest in northern temperate and polar regions. Late-century warming is much more widespread, especially under RCP 8.5, with substantial warming across much of the world’s EEZs.

Projected warming in SBT is lower, with mean warming under RCP 4.5 and 8.5 of 0.43–0.89 and 0.45–1.29°C, respectively, over mid- and late-century timescales (S5 Fig). The spatial imprint of predicted warming is similar to that for SST, though less strongly structured by latitude. Localised cooling is predicted in some areas, especially in the North Atlantic and the Arabian Gulf. As for SST, these patterns broadly intensify in the late century, especially under RCP 8.5.

### Species’ thermal affinities

Weighted and unweighted thermal affinities are similar across modelled species. For SST-derived thermal affinities, the mean and interquartile range of the differences between these two estimates for the three thermal affinity parameters (T_10_, STI and T_90_) ranges between -0.06–0.06 and 0.02–0.10°C respectively. The distribution of differences for SBT-derived thermal affinities is similar.

However, thermal affinities derived from SSTs are consistently warmer than those derived from SBTs, particularly for the lower thermal affinity (T_10_) and the STI (S6A and S6B Fig). For example, STIs derived from SSTs versus SBTs range between 3.19–28.36 (mean = 23.82) versus 1.45–20.09 (mean = 11.15)°C respectively. This difference is principally driven by species inhabiting areas in which SBTs are warmer than ~8°C, which cover an area over which SSTs are much more variable, ranging up to 30°C. For the upper thermal affinity (T_90_), the difference between SST- and SBT-derived thermal affinities is smaller, with SST-derived estimates 4.88°C higher on average compared to their SBT counterparts (S6C Fig). The net result is that STR estimates derived from SSTs are 7.79°C narrower on average than those derived from SBTs (S6D Fig).

### Species’ sensitivity

**Fig 2:**
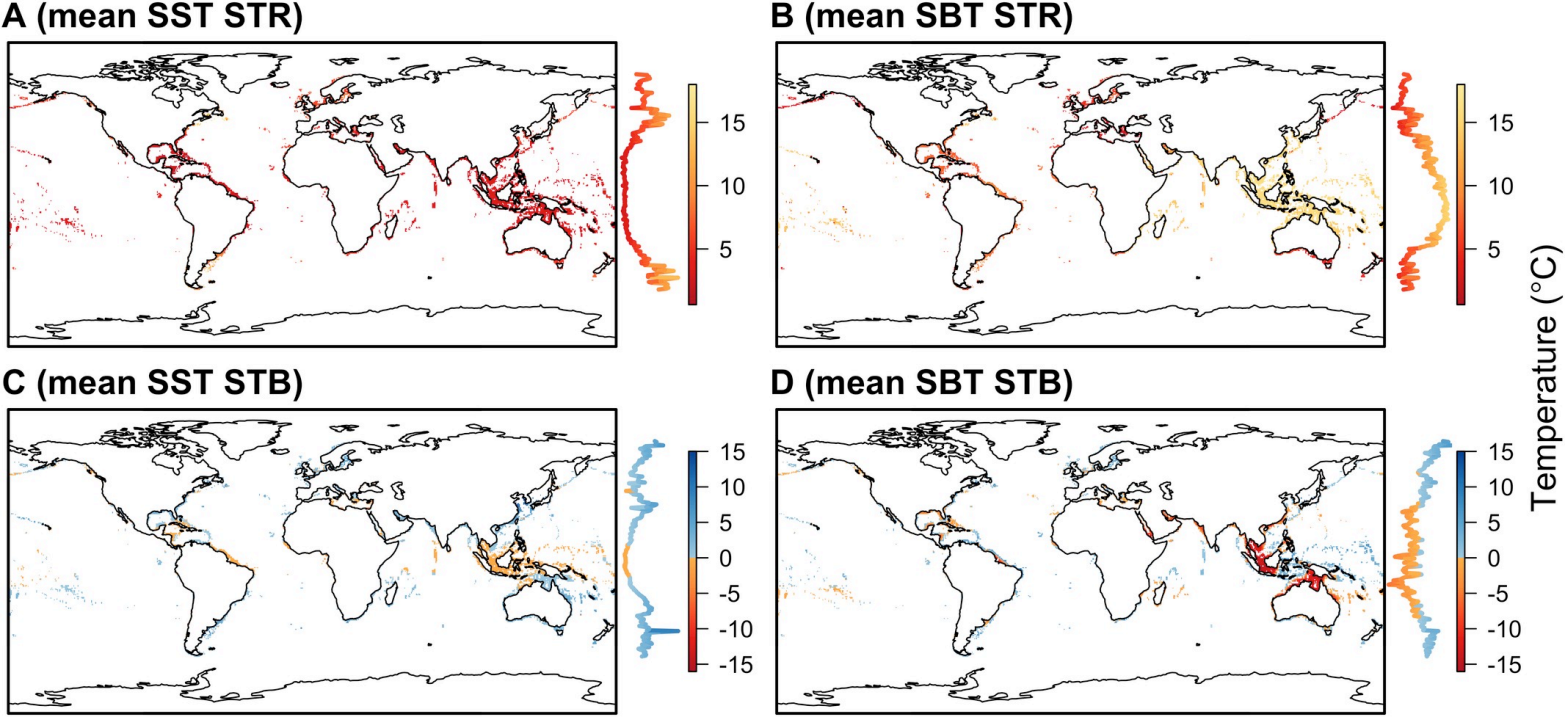
Spatial variation in species’ sensitivity to temperature change within Exclusive Economic Zones. A, B, The mean species’ thermal range (STR) across all species in each cell derived from (A) sea surface temperature (SST) and (B) sea bottom temperature (SBT). C, D, The mean species’ thermal bias (STB) derived from (C) SST and (D) SBT. Adjacent to each map, the coloured line shows the mean STR or STB across all cells in each latitudinal band, following same colour scheme as for the map. Background coastline data are from Natural Earth (public domain).

STRs track the spatial distribution of temperature gradients. SST-derived STRs are principally narrow in the tropics, particularly across the Indo-Pacific where the mean difference between the lower and upper thermal affinities is typically ≤ 5°C (Fig 2A). Wider STRs tend to be found in temperate regions, such as northern Europe and north-eastern Asia, southern South America and southern Oceania. Further north and south, STRs for modelled species narrow in association with more homogeneous temperature gradients in the polar regions. In contrast, SBT-derived STRs are widest in the Indo-Pacific (Fig 2B). In this region, the latitudinal turnover in SBT associated with transitions between shallow and deep water far exceeds that for SST. Consequently, species’ distributions in this region cover an area that is more variable in terms of SBT than SST, which increases the disparity between lower and upper thermal affinities. Thus, for shallow-water Indo-Pacific species, SBT appears less suitable for the estimation of thermal affinities than SST. Elsewhere, the distribution of SBT-derived STRs more closely resembles that for SST: in tropical and sub-tropical areas with more homogeneous temperature gradients, such as the Caribbean, STRs are relatively narrow, while in temperate regions, such as off Newfoundland, STRs are wider.

For SST-derived thermal affinities, the spatial organisation of species’ distributions and SSTs drives a strong latitudinal pattern in STBs (Fig 2C). In many tropical areas, especially in the Indo-Pacific, baseline temperatures are greater than inferred thermal optima on average (negative thermal bias). In Sundaland and Australasia, SBT-derived STBs are also most negative. However, in other equatorial regions, SBT-derived biases are less negative or even positive. Instead, SBT-derived biases are more negative along the tropics of Capricorn and Cancer, where SBTs are warmer. In temperate regions, both SST- and SBT-derived biases tend to be positive. These patterns are consistent across species, with the variability in STBs smallest in tropical regions and largest in temperate and polar regions (S7 Fig).

### Model predictions across a global grid

**Fig 3:**
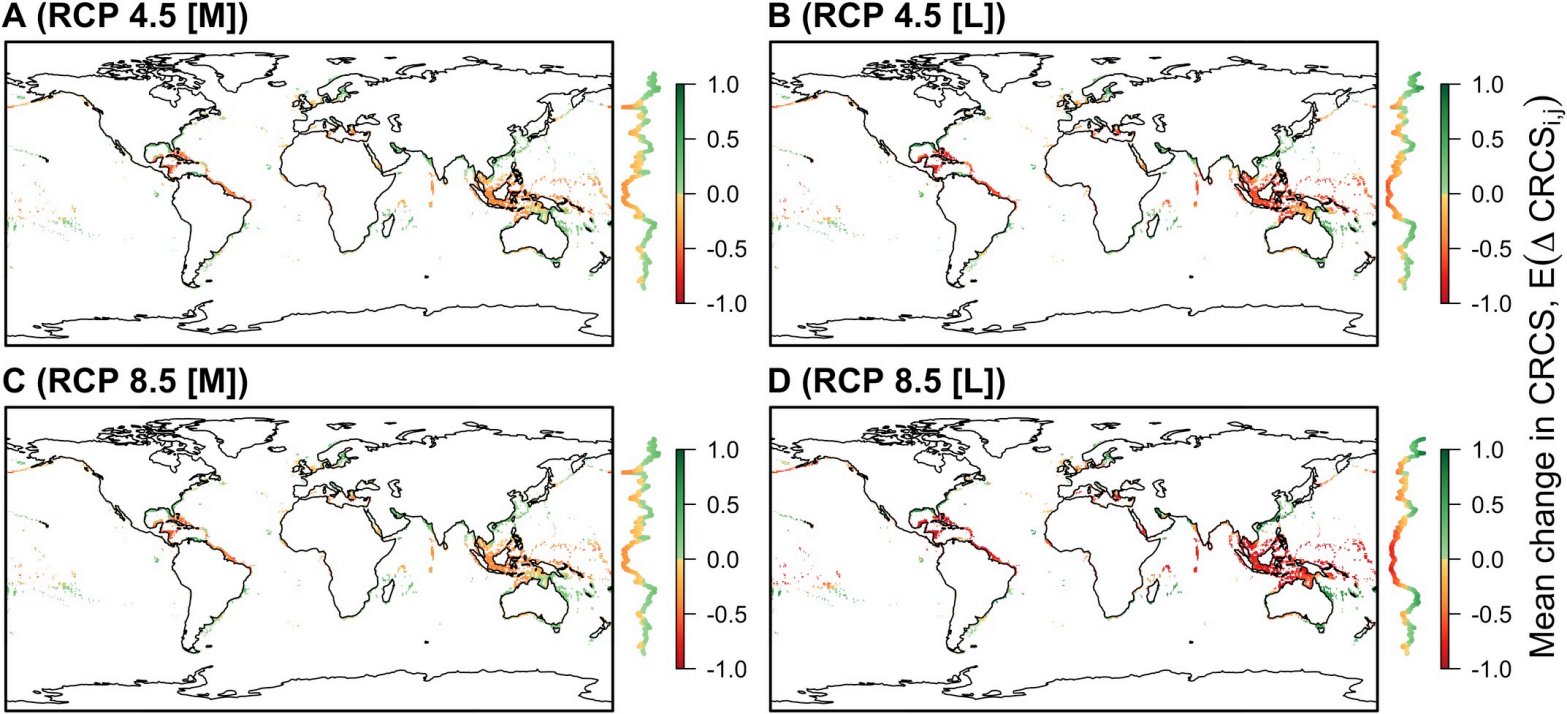
The mean predicted change in the index of thermal habitat suitability, E(ΔCRCS_i,j_), under future sea surface temperature (SST) change for two climate experiments (RCP 4.5 and 8.5) over mid-century (M) and late-century (L) timescales. In each 0.5° grid cell, the mean predicted change in the CRCS, calculated over all species whose predicted distributions overlap with that cell, is shown. Predictions are only shown within Exclusive Economic Zones. Adjacent to each map, the coloured line shows the mean E(ΔCRCS_i,j_) across all cells in each latitudinal band, following same colour scheme as for the map, with lower values (in red) to the left and higher values (in green) to the right. Background coastline data are from Natural Earth (public domain).

Predicted changes in the CRCS for modelled species under future SST experiments are strongly structured by latitude (Fig 3). In the tropics, predicted declines are most pronounced. The Indo-Pacific and the Caribbean stand out as the regions in which both species richness and the magnitude of predicted declines are highest. Even under RCP 4.5, in many tropical areas most modelled species are predicted to experience declines in CRCS by 2050 (S8 Fig). However, the magnitude of declines is predicted to intensify in the late century, especially under RCP 8.5.

In contrast, increases in CRCS are generally predicted with increasing latitude. In particular, high mean increases in CRCS are predicted in patches off North and South America and East Asia, despite more substantial temperature increases in these regions than in the tropics (Figs 3 and S4). However, fewer species in these regions were modelled relative to other regions (S2 Fig).

Under future SBT change, predicted declines in CRCS are also predicted in many tropical areas, though more spatially concentrated and typically weaker than for SST (S9 Fig). The most notable declines are predicted in Sundaland and Australasia, where STBs are negative. Declines are also predicted along the coastline of southern Asia, in the Caribbean, the Mediterranean and, to a lesser extent, in some inshore areas in the North Atlantic. However, elsewhere, small increases in CRCS are generally predicted.

### Model predictions across EEZs

**Fig 4:**
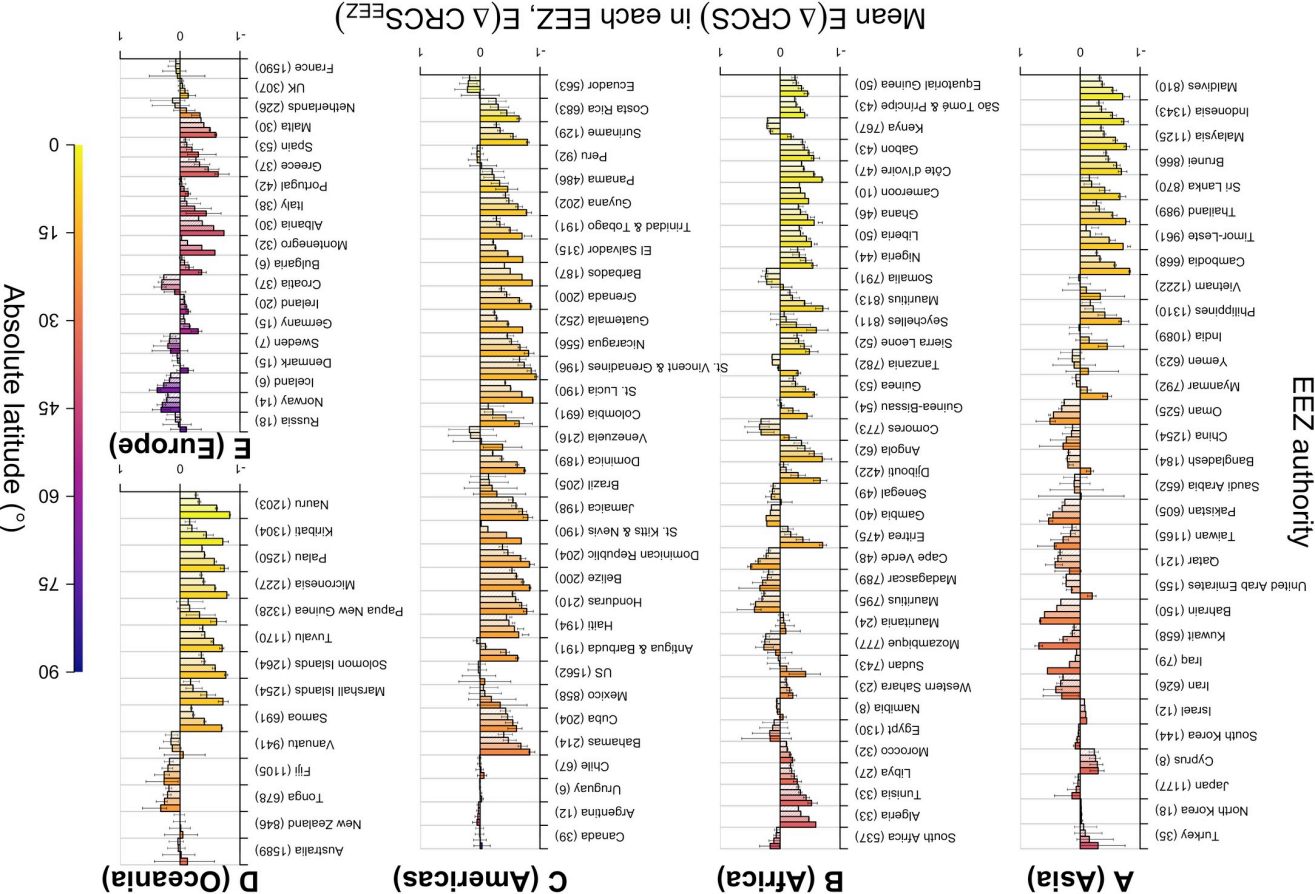
The mean predicted change in the index of thermal habitat suitability (CRCS) under future sea surface temperature change, synthesised across the coastal areas of all Exclusive Economic Zones (EEZs) containing more than five modelled species, in (A) Asia, (B) Africa, (C) the Americas, (D) Oceania and (E) Europe. For each EEZ, four bars are shown, denoting the average change in CRCS across all grid cells in that EEZ (E(ΔCRCS_EEZ_)), calculated from the mean predicted changes in each grid cell (E(ΔCRCS_i,j_)), under mid-century RCP 4.5 (bottom bar, lightest hatching) and RCP 8.5 (second bar) and late-century RCP 4.5 (third bar) and RCP 8.5 (top bar, densest hatching) experiments. For each EEZ, the colour of the top bar (the late-century RCP 8.5 experiment) indicates the EEZ’s (absolute) mid-point latitudinal location, which may be influenced by overseas territories. Error bars mark the interquartile range. Numbers in brackets after each EEZ indicate the number of modelled species.

**Fig 5:**
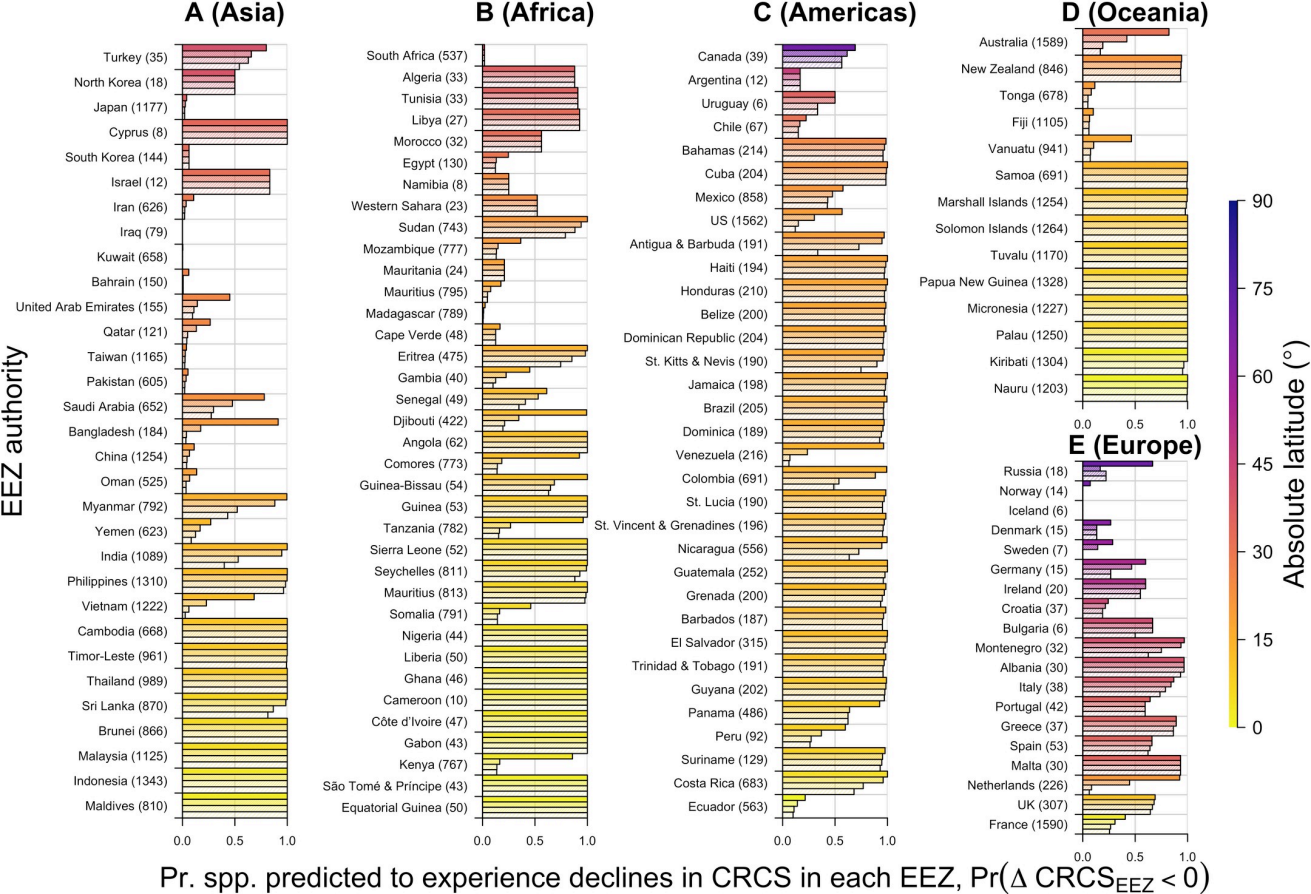
The proportion of species predicted to experience declines in the index of thermal habitat suitability (CRCS) under future sea surface temperature change in the coastal areas of all Exclusive Economic Zones (EEZs) containing more than five modelled species, in (A) Asia, (B) Africa, (C) the Americas, (D) Oceania and (E) Europe. For each EEZ, four bars are shown, denoting the proportion of species predicted to experience declines in CRCS in the EEZ (Pr(ΔCRCS_EEZ_ < 0)), under mid-century RCP 4.5 (bottom bar, lightest hatching) and RCP 8.5 (second bar) and late-century RCP 4.5 (third bar) and RCP 8.5 (top bar, densest hatching) experiments. For each EEZ, the colour of the top bar (the late-century RCP 8.5 experiment) indicates the EEZ’s (absolute) mid-point latitudinal location, which may be influenced by overseas territories. Error bars mark the interquartile range. Numbers in brackets after each EEZ indicate the number of modelled species.

For SST-based predictions, there are clear distinctions between EEZs that are expected to be ‘winners’ (where predicted changes in CRCS are largely positive) versus ‘losers’ (where predicted changes are largely negative; Figs 4 and 5). There is a clear dichotomy between low and high latitude EEZs. Most South East Asian EEZs represented by modelled species are predicted to be losers, including Malaysia, Thailand, Indonesia, the Philippines, India, Myanmar and Vietnam (Fig 4). For all of these EEZs, even under the mid-century RCP 4.5 experiment, most modelled species are predicted to experience declines in CRCS (Fig 5). However, the magnitude of predicted declines is more extreme in the late century, especially under RCP 8.5. In contrast, other Asian EEZs at higher latitudes, such as China, Taiwan, South Korea and Japan, are predicted to be winners, with most species predicted to increase in the CRCS.

Like South East Asia, almost all African EEZs are predicted to experience declines in the CRCS of modelled species on average (Figs 4 and 5). Predicted declines are especially severe in low latitude EEZs, such as Nigeria. In some higher latitude EEZs, predicted changes are smaller. For example, Morocco emerges as a marginal loser in terms of modelled species. Positive trends are only apparent in a handful of African EEZs, such as Madagascar and The Gambia.

Likewise, in most EEZs in North and South America represented by modelled species declines in CRCS are predicted (Figs 4 and 5). Declines in CRCS are especially pronounced in small nation states in the Caribbean. However, in other nations, such as the United States, predictions for modelled species are more variable. This pattern is repeated in Oceania, where predictions for small tropical and subtropical Pacific Island Countries, such as Samoa and the Marshall Islands, are consistently negative, while those for larger states, such as Australia and New Zealand, are more variable (Figs 4 and 5).

Predicted changes in the CRCS vary among European EEZs (Figs 4 and 5). In general, among these EEZs, predictions for the most southerly nations, such as Spain and Greece, are the most negative (Fig 4) and widespread across modelled species (Fig 5). In more northerly nations, such as France and the United Kingdom, weaker declines are predicted, but in most cases the proportion of modelled species predicted to experience declines in CRCS is still relatively high. There are only a few nations, such as Norway and Iceland, for which predictions are consistently positive. However, in these EEZs, predictions are driven by fewer shallow-water species.

### Comparison of climate experiments

**Fig 6:**
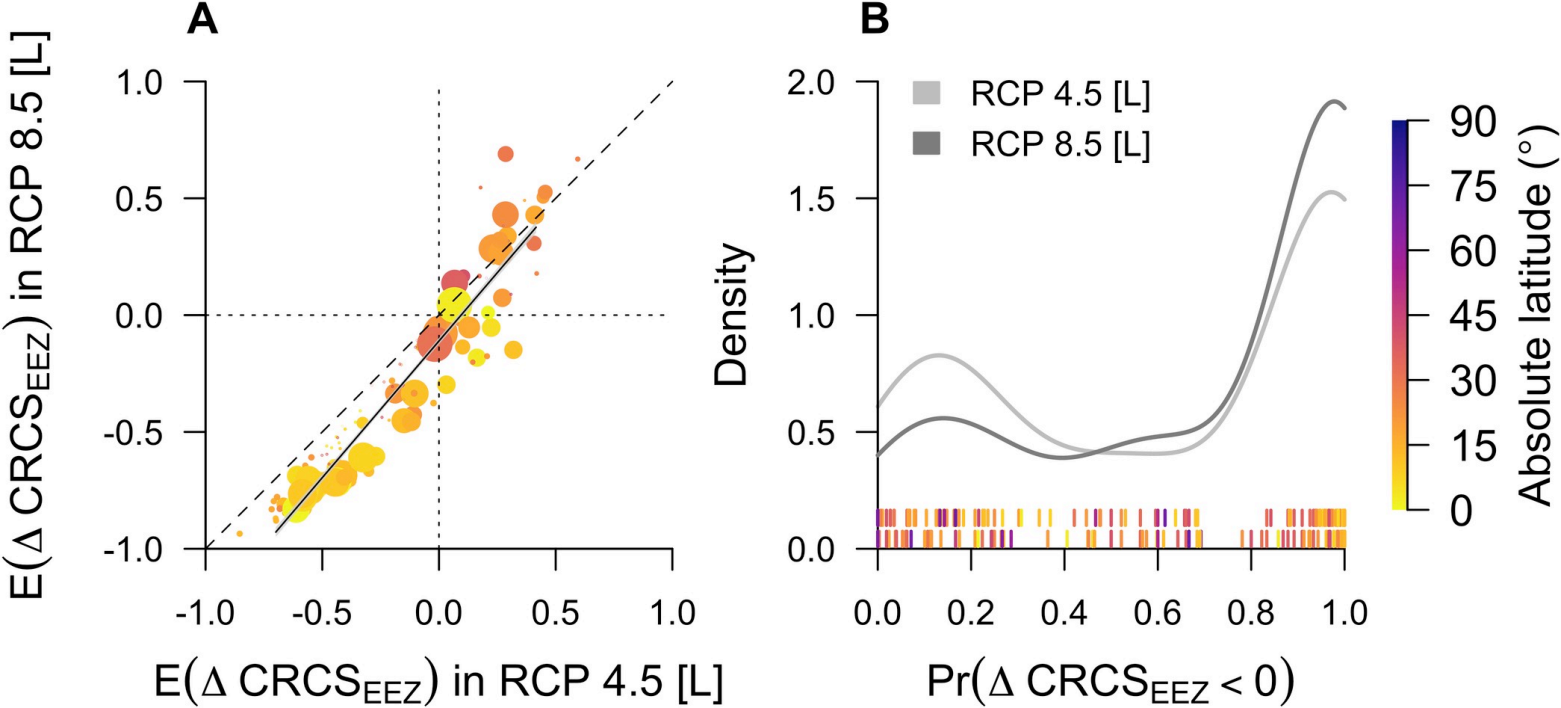
A comparison of predicted changes in the index of thermal habitat suitability (CRCS) between late-century climate experiments across Exclusive Economic Zones (EEZs). A, The mean predicted change in CRCS (E(ΔCRCS_EEZ_)) under RCP 8.5 as a function of the mean predicted change under RCP 4.5. B, Density distributions of the proportion of species predicted to experience declines in CRCS across EEZs (Pr(ΔCRCS_EEZ_ < 0)). In A, each point represents an EEZ; point size is proportional to the number of modelled species. The diagonal dashed line represents the line *y = x*. The solid black line surrounded by the grey confidence envelope marks the robust regression line ± 95% confidence intervals. The vertical and horizontal dotted lines mark a mean predicted change in CRCS of 0 under RCP 4.5 and 8.5 respectively. For EEZs below the line *y = x*, the predicted change in CRCS under RCP 8.5 is more negative than under RCP 4.5. For EEZs above the line, the predicted change in CRCS under RCP 8.5 is more positive. In B, the light and dark grey lines represent RCP 4.5 and 8.5 respectively. The upper and lower rugs mark the proportion of species predicted to experience declines in each EEZ under RCP 4.5 and 8.5 respectively. In both panels, EEZs are coloured according to their (absolute) mid-point latitudinal location.

Predicted changes in the CRCS of modelled species are broadly similar for the two experiments. For the mid-century time window, predicted declines are marginally greater under RCP 8.5 compared to RCP 4.5, particularly in the tropics, but the average difference in E(ΔCRCS_i,j_) for modelled species is only 0.03. Predicted declines are more widespread in the late century, especially for RCP 8.5, with an average difference in E(ΔCRCS_i,j_) between experiments for this time window of 0.17. Across EEZs, the estimate for the difference in E(ΔCRCS_EEZ_) under RCP 8.5 relative to RCP 4.5 is marginally negative (0.011 ± 0.009 standard error [s.e.]; *t* = 11.68) and the gradient is slightly greater than one (1.170 ± 0.024 s.e.; degrees of freedom [df] = 131; Fig 6A). Thus, EEZs with declines, on average, in CRCS under RCP 4.5 are expected to experience greater declines under RCP 8.5, but this effect is small.

The density distribution of the proportion of species predicted to experience declines in CRCS across EEZs by the late century is bimodal in shape for both experiments (Fig 6B). There are relatively few EEZs where the proportion of species predicted to experience declines is low. Under RCP 4.5, only one quarter of EEZs (*n* = 34/133), are predicted to experience declines across less than 20% of species and in half of the EEZs represented (*n* = 69/133), declines across more than 80% of species are predicted.

## Discussion

Our results suggest that climate change is likely to have widespread impacts on thermal habitat suitability for shallow-water marine fish over the 21^st^ century. For modelled species, sensitivity to projected changes in SST peaks in the tropics where STRs are narrowest and STBs are consistently negative. For the index of thermal habitat suitability (CRCS), this translates into a clear pattern, with widespread declines in CRCS predicted across inshore tropical areas and contrasting increases at higher latitudes. This pattern is broadly reflected in the EEZs that are predicted to be winners and losers and is likely to drive large-scale changes in the relative abundance of shallow-water fish, in-line with the predictions of previous global-scale modelling studies that have focused on other marine assemblages [28, 39–43]. Many of the same patterns emerge from predictions based on SBT, though this appears to be less suitable for characterising shallow-water thermal affinities, despite the fact that some modelled species may experience temperatures that differ from SST. Taken together, these results add to the evidence that future temperature change is likely to have major impacts in coastal marine ecosystems, including for many species that are not commerically targeted. Alongside the pursuit of large-scale reductions in greenhouse gas emissions, this highlights the urgent need to strengthen ecosystem-based management and adaptive capacity in coastal communities, particularly those in the tropics that are most vulnerable to climate change [12, 20].

Spatial patterns in the distribution of species’ thermal affinities underlie species’ sensitivities to future temperature change. As in other studies [56, 66, 87, 88], in the tropics we find that STRs estimated from the quantiles of variation in SST across species’ distributions tend to be narrower and negative STBs predominate, which may make species vulnerable to relatively small temperature increases. In contrast, in temperate regions, STRs tend to be wider, which means that assemblage-wide declines in thermal habitat suitability with temperature change are less likely [58]. Furthermore, large, positive STBs at higher latitudes make many of these species resilient to even relatively large temperature increases.

In line with species’ sensitivities, we identify a strong latitudinal pattern in predicted changes in the CRCS of modelled species with future changes in SST. Widespread declines are predicted across most shallow-water tropical marine fish species that we modelled, while increases in the CRCS are predicted for temperate species. These predictions emerge despite a disparity in the magnitude of projected SST changes between low- and high-latitude regions, highlighting the importance of species’ thermal affinities in modulating the effects of future temperature change. Changes in the CRCS for modelled species across coastal areas are broadly reflected in the EEZs predicted to be winners and losers. Across the board, low-latitude nations in the Indo-Pacific, the Caribbean and across much of Africa emerge as areas of concern, where predictions are consistently negative for modelled species. Even under the ‘intermediate’ climate experiment, in many of these locations nearly all modelled species are predicted to decline in their CRCS before 2050, though the magnitude of predicted declines is greater over longer timescales, especially under the ‘business-as-usual’ climate experiment. In contrast, predictions for many higher latitude nations are more variable, with some nations, such as Norway, emerging as possible beneficiaries of future temperature change, at least in terms of shallow-water fish assemblages. For some nations, there is a disparity between the predictions for the regions in which they are situated and the coastal areas under their jurisdiction. For example, the high proportion of species predicted to experience declines in CRCS in the United Kingdom compared to other nations at a similar latitude is driven by the relatively high number of British Overseas Territories, many of which lie in the tropics.

Four main sources of uncertainty caveat our predictions. The first source of uncertainty comes from the use of Aquamaps SDMs to describe species’ distributions and estimate thermal affinities. The advantage of this choice is that Aquamaps SDMs are based on a standardised methodology that has been widely applied to marine species, including many relatively data-poor tropical and subtropical species [44, 51]. However, Aquamaps SDMs are uncertain and predictive accuracy varies [44, 51, 83]. In a validation study based on 12 species, Aquamaps SDMs only correlated significantly with independent survey data in 30–50% of cases, though they compared favourably with alternative approaches [44]. A key parameter is the number of unique grid cells that contain valid occurrences (i.e. occurrence observations within a species’ known natural range) [44, 51, 83]. This parameter is important as a measure of the number of observations used for model fitting. A comparison of Aquamaps SDMs and range maps from the International Union for Conservation of Nature (IUCN) demonstrated reasonable agreement between the two approaches, particularly for well-studied species with a median of 41 unique cells with valid occurrences [83]. The worst agreement occurred for species with a median of 10 unique cells with valid occurrences. For this reason, in our analysis, we only considered species with more than 10 unique cells with valid occurrences and the majority of modelled species (n = 1,434, 62.54%) had more than 41 unique cells with valid occurrences. Nevertheless, even for more data-rich species, Aquamaps SDMs are uncertain, especially along range edges where the influence of Major Fishing Area boundaries to constrain species’ ranges can induce discontinuities [83]. The discretisation of SDMs into binary presence/absence maps introduces an additional source of uncertainty that can affect range boundaries [83]. For individual SDMs, these uncertainties are particularly important. For this reason, we used the 10^th^, 50^th^ and 90^th^ quantiles in temperature as estimates of species’ thermal affinities: these statistics are relatively robust to uncertainty in SDMs, as shown by the comparison of unweighted and weighted thermal affinities. Beyond individual SDMs, for global-scale studies the overall patterns that emerge from overlaying SDMs for many species are likely to be much more robust to uncertainties in the edges of species’ distributions [89, 90]. However, uncertainty in SDMs is a common caveat for global-scale modelling studies and continued efforts to support data collection and develop ensemble modelling frameworks will support future research in this area.

The second source of uncertainty lies in nature of species’ thermal affinity estimates, which depend on the assumption that the ambient thermal experience of modelled species is effectively described by (a) the selected temperature variable and (b) spatiotemporal scale over which estimates are derived. To support the first assumption (a), we focused on shallow-water species because their ambient thermal environment is likely to be well-described by SSTs, for which projections are widely available and applicable. The restriction of ‘shallow-water’ species to those that exclusively inhabit water above 50 m reflects the compromise between ensuring that species’ thermal affinities are reasonably accurate whilst retaining a sufficient number of species to model change at a global scale. While shallow-water species may experience temperatures that differ from SSTs, particularly in areas with shallow-water thermoclines [91], the average depth of modelled species suggests that this choice was broadly appropriate. For future research, a question remains regarding how to estimate the thermal affinities of species that experience a range of temperatures throughout the water column. However, for shallow-water species we find SBT to be less appropriate for the characterisation of thermal affinities. Unlike SST, available one-degree SBT projections are strongly associated with bathymetry, which drives sharp differences between shallow and deep areas that do not reflect the depths inhabited by modelled species. In places such as the Indo-Pacific, this gives rises overly wide STRs and switches in STBs, resulting in contrasting predictions between nearby shallow and deep areas. Nevertheless, elsewhere, it is notable that, in many areas, spatial gradients in SST and SBT—and the resultant pattern of predictions for CRCS—are similar, though predicted changes based on SBT are smaller.

For both SST and SBT, a related issue (b) that is relevant to most global-scale modelling studies is the potential mismatch between the temperatures that individuals experience and the long-term average temperatures used to derive thermal affinities and make predictions. While there are multiple temperature metrics that can affect fish in marine environments [92] and thermal affinities estimated across species’ distributions may mask spatial variability resulting from local adaptation [93], there is robust evidence that thermal habitat suitability is associated with long-term temperature variation across broad spatiotemporal scales [29, 31, 33, 58]. For some species, local thermal affinities may be better specific predictors of climatic impacts, but in the absence of information on population structure, distribution-wide estimates are reasonable approximations which should effectively capture the extent to which species are more or less sensitive to temperature change on average [36, 70]. Likewise, other temperature metrics may be better specific predictors of climatic impacts, but these are likely to correlate with the average temperature, which is most widely applicable. For example, a principle driver of marine heatwaves is long-term climate change [94, 95] and thermal affinities derived from long-term average annual SSTs can successfully predict the response of communities to these, at least in some circumstances [49, 96]. Species’ responses to extreme conditions may be larger than those expected from decadal warming [81], and warrant further research, but take place against the backdrop of longer-term changes.

The third source of uncertainty stems from the use of species’ climate response curves to represent thermal habitat suitability. In support of this concept, both theory and empirical evidence demonstrate the critical influence of temperature on the physiology of marine organisms, with temperatures away from thermal optimal leading to a decline in aerobic performance [4]. Recent empirical evidence suggests that this relationship can be parameterised from realised species’ distributions [49, 56, 97]. A global-scale analysis of rocky and coral reef species supported the hypothesis that the STI represents the temperature of maximum thermal habitat suitability, revealing a reasonable correspondence between the STI and the temperature of maximum local abundance [56]. Similarly, in a study which compared the ability of the STI, body size and range size to explain species-level responses to extreme temperature variation on coral reefs in Australia, the STI emerged as the strongest predictor of species-level responses in presence/absence and abundance [49]. Beyond the STI, warming temperatures are widely associated with declines in thermal habitat suitability (apparent from declines in abundance) at warm range edges and increases in thermal habitat suitability (apparent from increases in abundance) at cool range edges [34, 67, 68]. Collectively, these studies provide compelling support for the utility of climate response curves.

A key assumption in our approach is the use of a Gaussian function to represent climate response curves. An advantage of this choice is that few data are needed to parameterise a Gaussian function, making the approach applicable across a wide range of species. In line with this assumption, evidence from eight important commercial species in Europe showed that the distribution of abundance with respect to temperature is at least triangular in shape [98]. Furthermore, Gaussian climate response curves were a reasonable model for many of the reef fishes evaluated by Waldock *et al*. [57] and for the fish and plankton species modelled by Burrows *et al*. [58], emerging as the most applicable model on average across many species. However, climate response curves can take other shapes [38, 46, 55, 57]. Bonachela *et al*. [46] examine the importance of species-specific skewness. Empirical evidence suggests that skewness increases towards the poles and tropics, with some temperate species exhibiting cold-skewed distributions and tropical species exhibiting warm-skewed distributions [57]. For modelling the impacts of climate change, warm skew among tropical species is of most concern because it implies that even small temperature increases could lead to drastic declines in thermal habitat suitability. However, the extent to which warm skew reflects the use of SDMs to infer thermal affinities (which constrains temperate and tropical species’ thermal affinities to the minimum and maximum baseline temperatures respectively) or physiological tolerances remains uncertain. Recent work indicates that species richness has declined around the equator in line with temperature increases [99]. While this fits with the direction of future changes in thermal habitat suitability around the equator that we predict using Gaussian climate response curves, the magnitude of those changes may be more or less severe depending on the extent to which species can tolerate temperatures beyond the current global maximum temperatures. This is an important knowledge gap for future research.

The fourth source of uncertainty lies in the extent to which populations will respond to changes in thermal habitat suitability. We focused our analysis on changes within the current range limits of modelled species. Beyond these limits, analyses of climate velocities suggest that range shifts are generally relatively small in comparison to the size of EEZs [67, 100]. Nevertheless, for some species, the redistribution of individuals beyond current range limits may partly counteract unfavourable changes within existing ranges. Our application also assumed that thermal affinities are temporally stationary. Available evidence suggests that the pace of current temperature change is substantially greater than the pace of adaptive evolution [1], indicating that this assumption is likely to be well met for many species over the next few decades. However, over longer timescales, *in situ* adaptation may help to mitigate some of the impacts of temperature change.

Despite these uncertainties, the broad-scale patterns that we predict for changes in the thermal habitat suitability of shallow-water fish based on climate response curves are clear. Furthermore, the links between thermal habitat suitability and abundance imply that predicted changes in thermal habitat suitability are likely to correspond in broad terms with changes in abundance, with declining thermal habitat suitability associated with declines in abundance and increases in thermal habitat suitability associated with increases in abundance [29, 31]. These expectations are consistent with other global-scale modelling studies [28, 39–43]. For example, in line with our predictions, a dynamic climate envelope modelling approach, in which the effects of climate change are primarily realised through temperature-driven shifts in species’ ranges, predicts declines in catch potential of up to 40% in the tropics and increases of 30–70% at high latitudes [40]. Dynamic, size-based food web models, in which the effects of climate change are realised through temperature effects on feeding and intrinsic mortality rates in size-structured communities, and ensemble global ecosystem model approaches, also predict similar patterns at large spatial scales [28, 41, 43]. We view our approach as a complement to these models—a tool to predict large-scale patterns in the direction of change in the CRCS across poorly known species. These predictions are likely to correlate with the direction of change in abundance, but absolute change in abundances are likely to be dependent on many localised factors, such as density dependence, not captured by our model [101].

The convergence of modelling approaches on large-scale patterns has important implications for understanding climate vulnerability, mitigation and adaptation. Healthy fish populations structure ecosystems, supporting ecosystem functioning and the delivery of ecosystem services, such as fisheries [6, 10, 11]. The correspondence between predictions for tropical areas is especially concerning given their diversity, the strong reliance of many tropical communities on subsistence inshore fisheries, existing patterns of overexploitation and limited adaptive capacity [12, 20]. While the absence of data on the importance of most of our modelled species to humans suggests that they are not commerically targeted, in many tropical and subtropical areas, the use of fine-mesh nets and destructive fishing techniques is associated with the exploitation of a large number of species that go unrecorded in official fisheries statistics [102–104]. In the Solomon Islands (Human Development index: 0.569), subsistence fisheries far surpass commercial fisheries in importance, with catches of 15,000 metric tonnes (mt) versus 3,250 mt per year respectively [105]. Healthy fish populations also support alternative sources of income, such as marine ecotourism [106, 107]. Yet many coastal fish populations are already under pressure, and worsening conditions may further reduce compliance with regulations and promote the use of increasingly destructive fishing techniques in a downwards spiral [108]. The strong declines in thermal habitat suitability that we predict for tropical inshore areas under future temperature change add to this concerning picture. In this context, the need to discriminate between declines due to overexploitation and those due to climate change, and to improve inshore fish stock assessments and regulation, is paramount for successful ecosystem-based management [109].

There is increasing evidence that strong fisheries management and marine reserves can rebuild stocks and offset some of the negative impacts of climate change [23, 110]. For example, improving catch selectivity and reducing fishing pressure can benefit both targeted and non-targeted species [111]. Larger populations in less disturbed areas may be more resilient to climate change and can help to sustain local fisheries [112, 113]. At the same time, marine reserves that effectively protect coastal ecosystems can offer additional benefits, such as flood mitigation, which can help communities adapt to changing conditions [114]. However, for many tropical and sub-tropical jurisdictions, the development and implementation of strong fisheries advice and enforcement in the context of weak financial and governance institutions remains a continuing challenge [20]. Moreover, ultimately, local and regional ecosystem-based management approaches must be accompanied by global emissions reductions to address the climate emergency and mitigate its impacts on coastal communities [110].

## Supporting information

S1 Fig Quantiles of observed variation in the shallow and deep depth range limits of modelled species. Shallow depth quantiles represent 2,150 species with data and deep depth quantiles represent all 2,293 species.
(TIF)

S2 Fig The spatial distribution of modelled species across a global grid at 0.5° resolution. Cells shown in white represent no data. Background coastline data are from Natural Earth (public domain).
(TIF)

S3 Fig The spatial distribution of baseline (1956–2005) temperatures within Exclusive Economic Zones at 0.5° resolution for (A) sea surface temperature (SST) and (B) sea bottom temperature (SBT). Background coastline data are from Natural Earth (public domain).
(TIF)

S4 Fig Future sea surface temperature (SST) projections within Exclusive Economic Zones at 0.5° resolution. Each plot shows the projected change in temperature, relative to the baseline, under a specific climate experiment (RCP 4.5 or RCP 8.5) over mid-century (M) or late-century (L) timescales. Positive numbers indicate warming. Background coastline data are from Natural Earth (public domain).
(TIF)

S5 Fig Future sea bottom temperature (SBT) projections within Exclusive Economic Zones at 0.5° resolution. Each plot shows the projected change in temperature, relative to the baseline, under a specific climate experiment (RCP 4.5 or RCP 8.5) over mid-century (M) or late-century (L) timescales. Negative numbers indicate cooling (shown in blue) and positive numbers indicate warming (shown in orange/red). Background coastline data are from Natural Earth (public domain).
(TIF)

S6 Fig Correlations between thermal affinities derived from baseline sea surface temperature (SST) and sea bottom temperature (SBT) projections. A, the lower thermal affinity (T_10_); B, the species’ thermal index (STI); C, the upper thermal affinity (T_90_); and D, the species’ thermal range (STR). In each plot, each point represents a modelled species. The line y = x is shown to aid interpretation.
(TIF)

S7 Fig Spatial patterns in the variability of species’ thermal biases (STBs) within Exclusive Economic Zones at 0.5° resolution, derived from baseline (A) sea surface temperature (SST) and (B) sea bottom temperature (SBT) projections. In each grid cell, the interquartile range (IRQ) in thermal bias, calculated over all species whose predicted distributions overlap with that cell, is shown. Adjacent to each map, the coloured line shows the mean IQR across all cells in each latitudinal band, following same colour scheme as for the map. Background coastline data are from Natural Earth (public domain).
(TIF)

S8 Fig The proportion of species predicted to experience declines in the index of thermal habitat suitability, Pr(ΔCRCS_i,j_ < 0), under future sea surface temperature (SST) change for two climate experiments (RCP 4.5 and 8.5) over mid-century (M) and late-century (L) timescales. In each 0.5° grid cell, the proportion of species predicted to experience decline in the CRCS, out of the total number of species whose predicted distributions overlap with that cell, is shown. Predictions are only shown within Exclusive Economic Zones. Adjacent to each map, the coloured line shows the mean Pr(ΔCRCS_i,j_ < 0) across all cells in each latitudinal band, following same colour scheme as for the map, with lower proportions (in green) to the left and higher proportions (in red) to the right. Background coastline data are from Natural Earth (public domain).
(TIF)

S9 Fig The mean predicted change in the index of thermal habitat suitability, E(ΔCRCS_i,j_), under future sea bottom temperature (SBT) change for two climate experiments (RCP 4.5 and 8.5) over mid-century (M) and late-century (L) timescales. In each 0.5° grid cell, the mean predicted change in the CRCS, calculated across all species whose predicted distributions overlap with that cell, is shown. Predictions are only shown within Exclusive Economic Zones. Adjacent to each map, the coloured line shows the mean E(ΔCRCS_i,j_) across all cells in each latitudinal band, following same colour scheme as for the map, with lower values (in red) to the left and higher values (in green) to the right. Background coastline data are from Natural Earth (public domain).
(TIF)

S1 Table A list of modelled species. Taxa are ordered by alphabetically by order, class, family and genus. For each species, the number of unique 0.5° cells with occurrence observations used by Aquamaps to generate the species’ distribution is shown. Cell occurrence counts vary between 10–1015 (median = 58, mean = 96, standard deviation = 103) cells. The 10^th^, 50^th^ and 90^th^ quantiles in baseline sea surface temperature across species’ distributions (rounded to two decimal places) are also provided.
(TXT)
