# Supplementary figures and images for: Modelling the impacts of climate change on thermal habitat suitability for shallow-water marine fish at a global scale
Source: PLoS One. 2021 Oct 4;16(10):e0258184. doi: 10.1371/journal.pone.0258184 (PMC8489719; doi:10.1371/journal.pone.0258184)

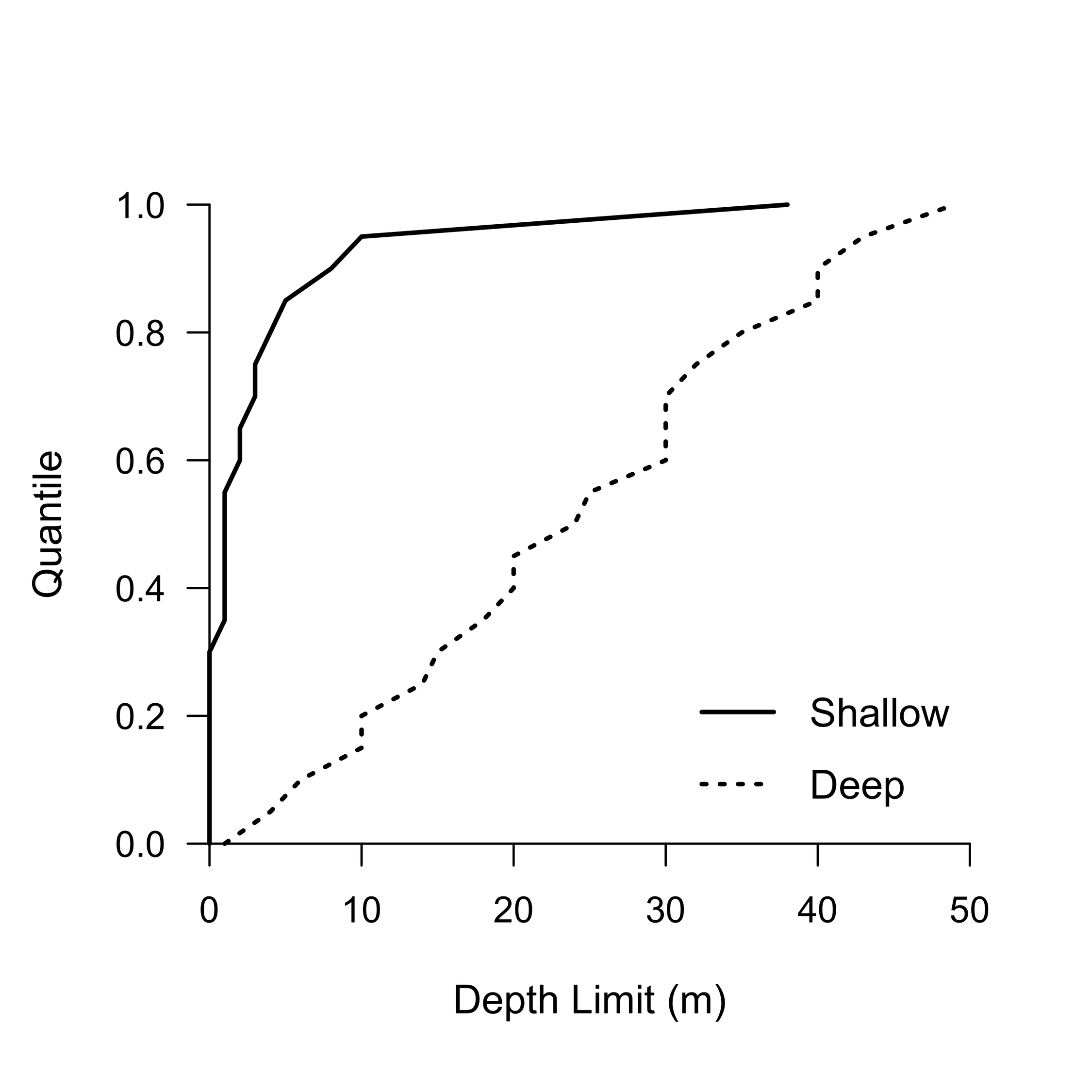

Supplement: S1 Fig — Shallow depth quantiles represent 2,150 species with data and deep depth quantiles represent all 2,293 species. (TIF) [file pone.0258184.s001.tif]

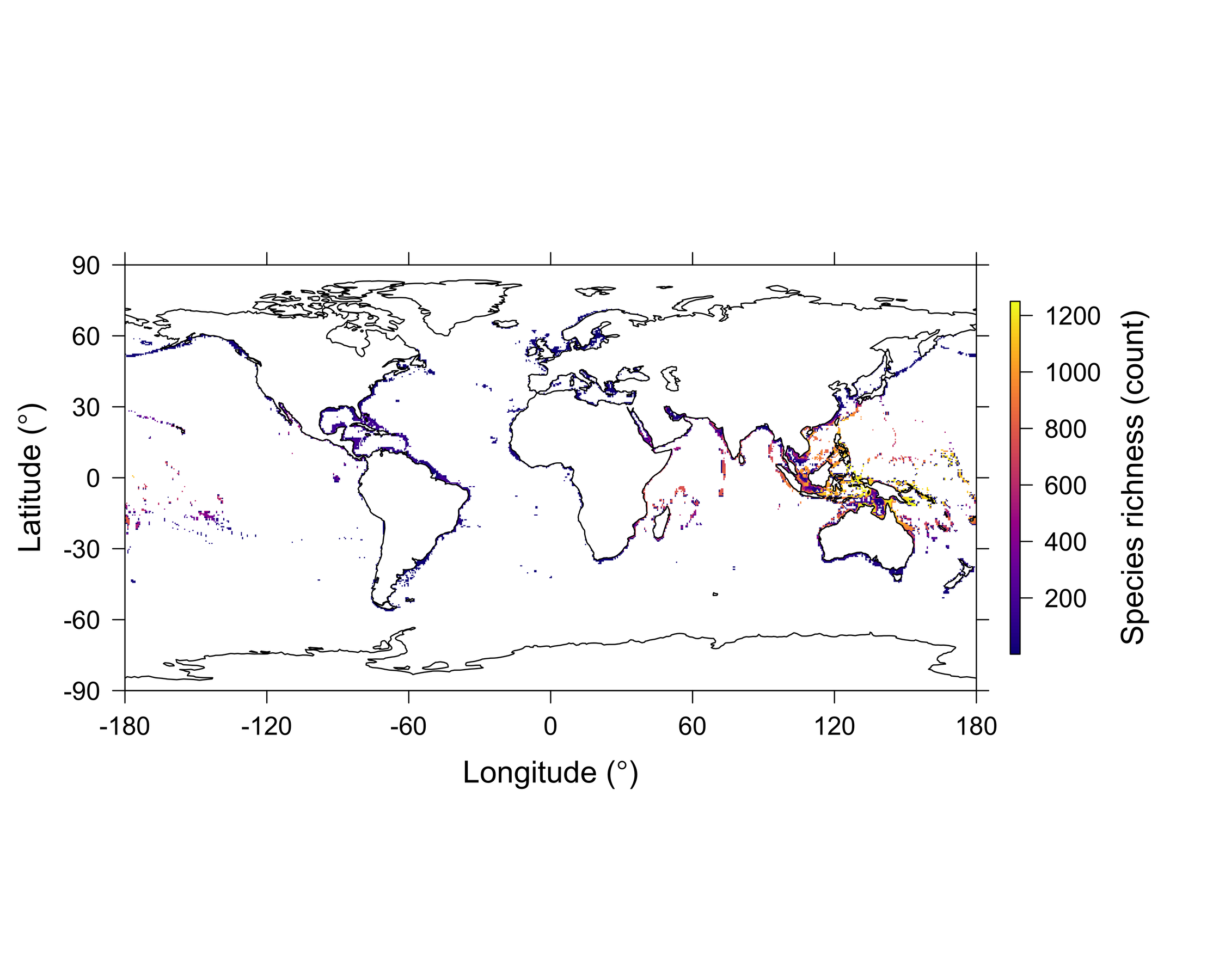

Supplement: S2 Fig — Cells shown in white represent no data. Background coastline data are from Natural Earth (public domain). (TIF) [file pone.0258184.s002.tif]

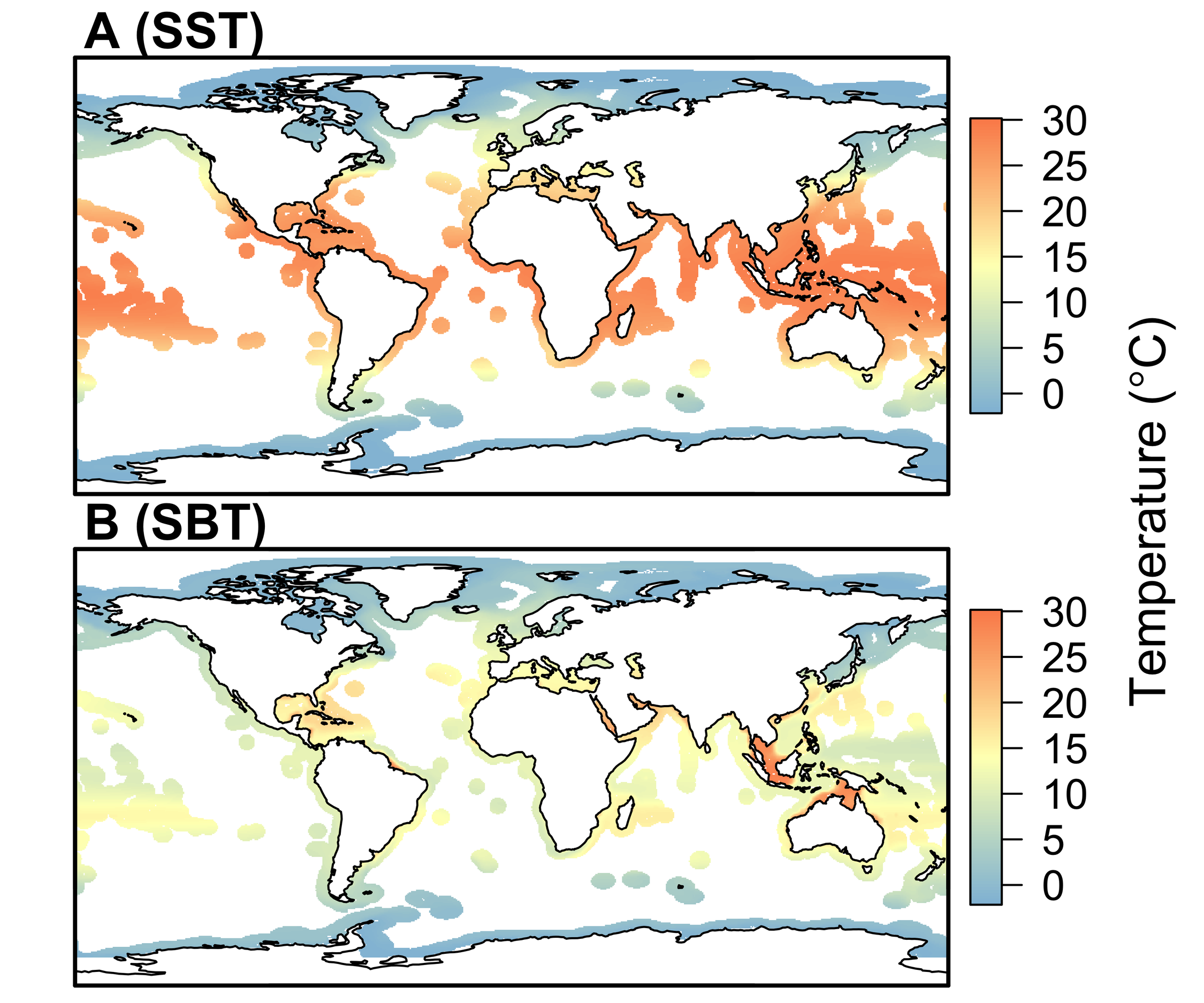

Supplement: S3 Fig — The spatial distribution of baseline (1956–2005) temperatures within Exclusive Economic Zones at 0.5° resolution for (A) sea surface temperature (SST) and (B) sea bottom temperature (SBT). Background coastline data are from Natural Earth (public domain). (TIF) [file pone.0258184.s003.tif]

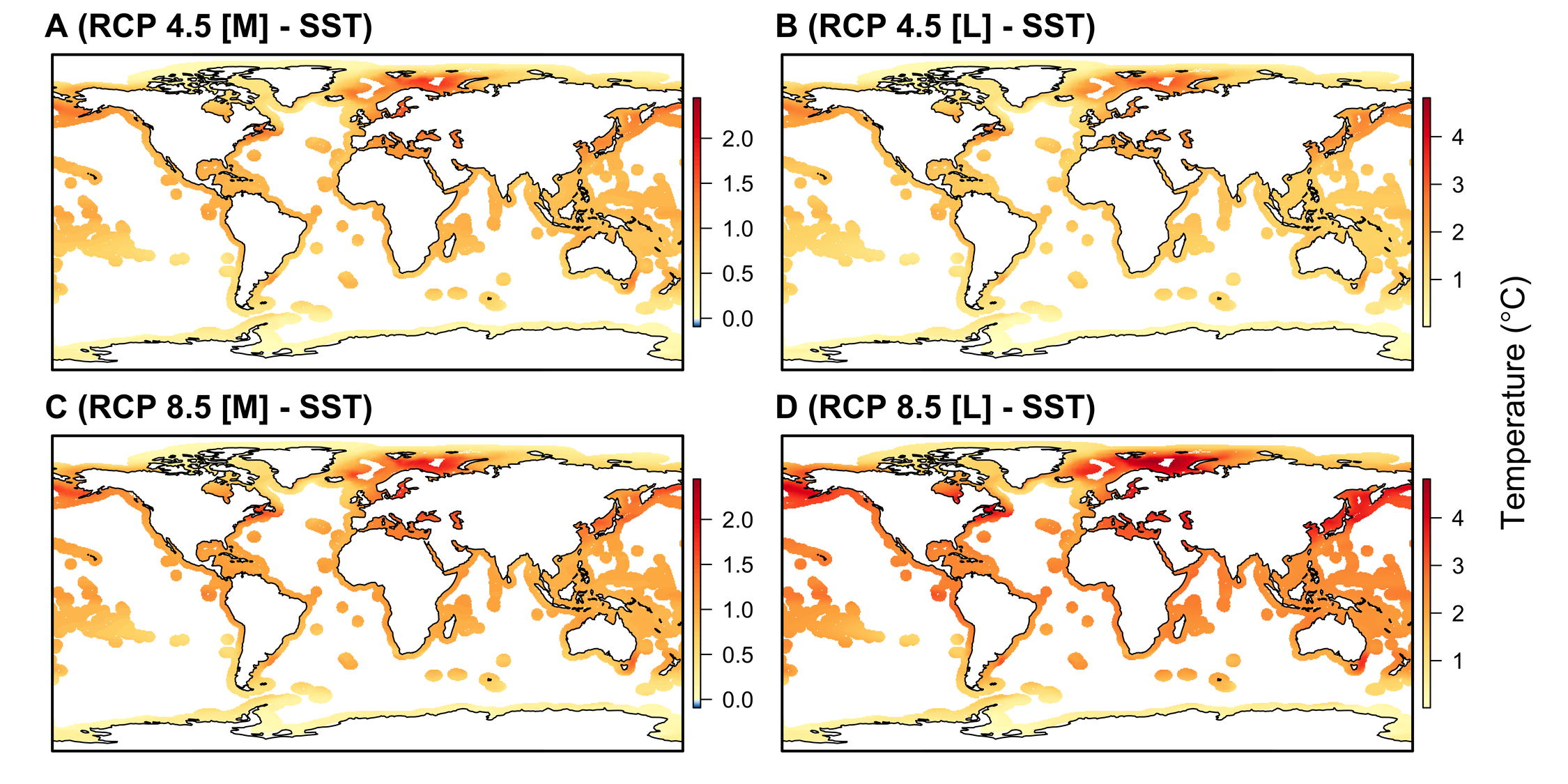

Supplement: S4 Fig — Each plot shows the projected change in temperature, relative to the baseline, under a specific climate experiment (RCP 4.5 or RCP 8.5) over mid-century (M) or late-century (L) timescales. Positive numbers indicate warming. Background coastline data are from Natural Earth (public domain). (TIF) [file pone.0258184.s004.tif]

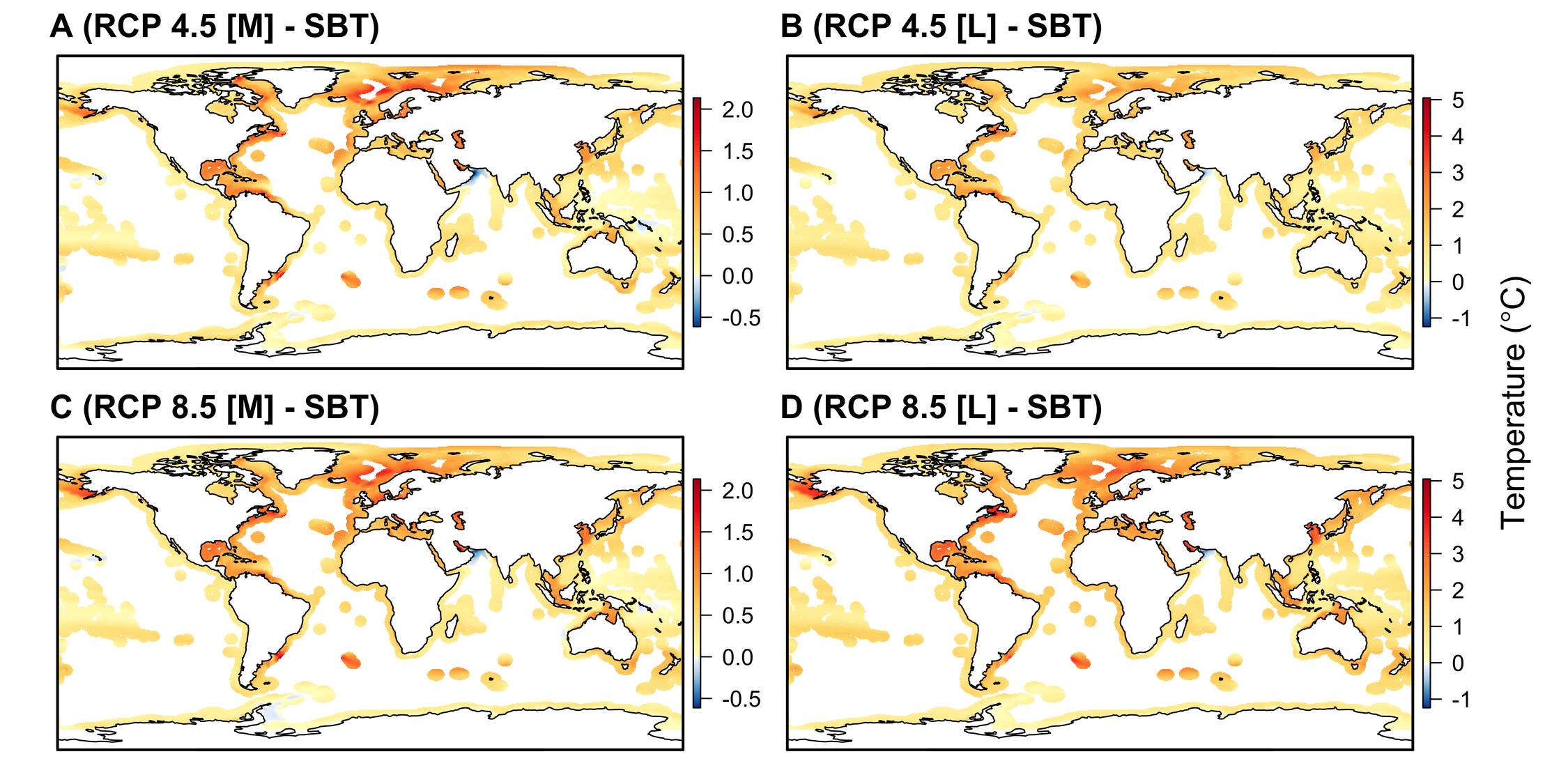

Supplement: S5 Fig — Each plot shows the projected change in temperature, relative to the baseline, under a specific climate experiment (RCP 4.5 or RCP 8.5) over mid-century (M) or late-century (L) timescales. Negative numbers indicate cooling (shown in blue) and positive numbers indicate warming (shown in orange/red). Background coastline data are from Natural Earth (public domain). (TIF) [file pone.0258184.s005.tif]

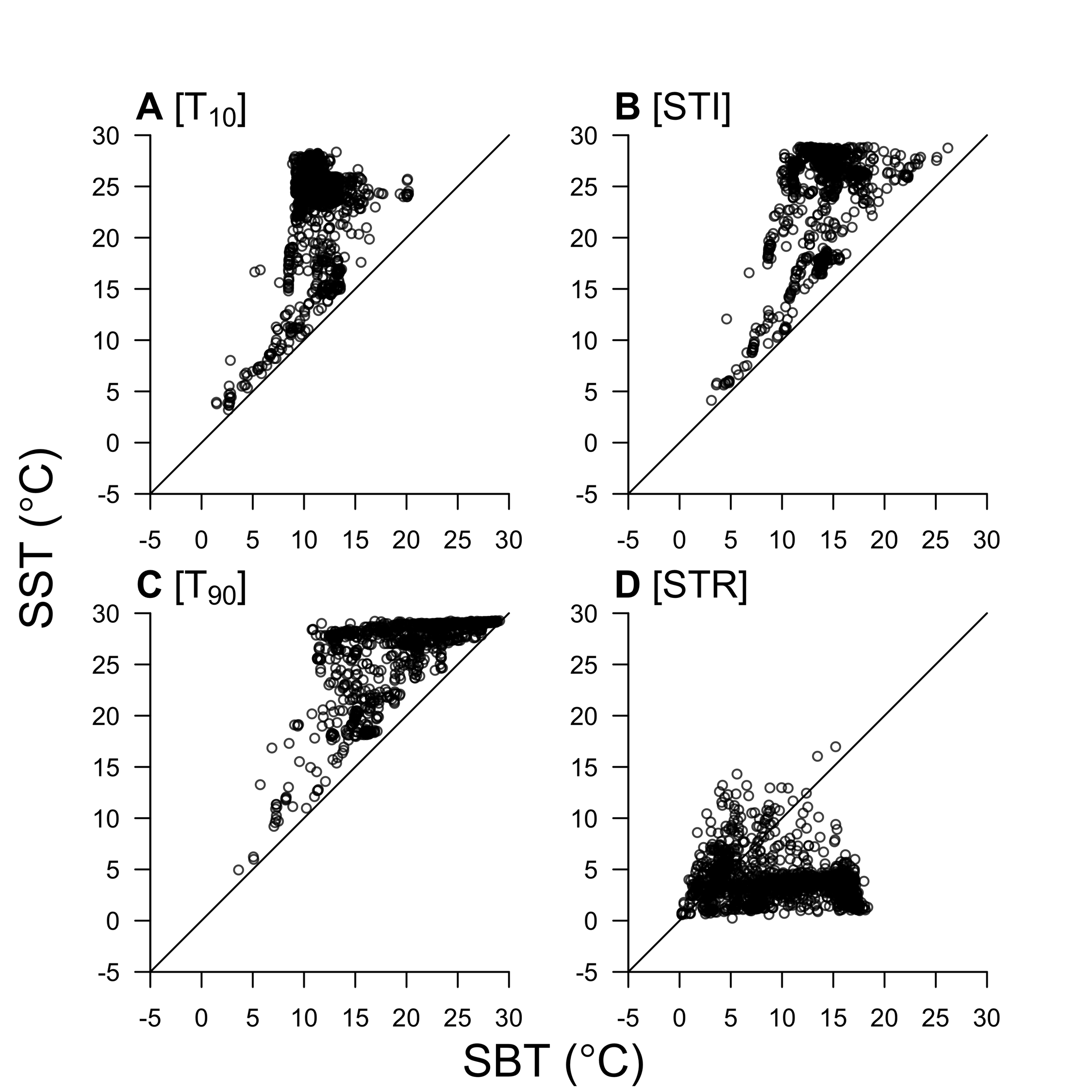

Supplement: S6 Fig — A, the lower thermal affinity (T10); B, the species’ thermal index (STI); C, the upper thermal affinity (T90); and D, the species’ thermal range (STR). In each plot, each point represents a modelled species. The line y = x is shown to aid interpretation. (TIF) [file pone.0258184.s006.tif]

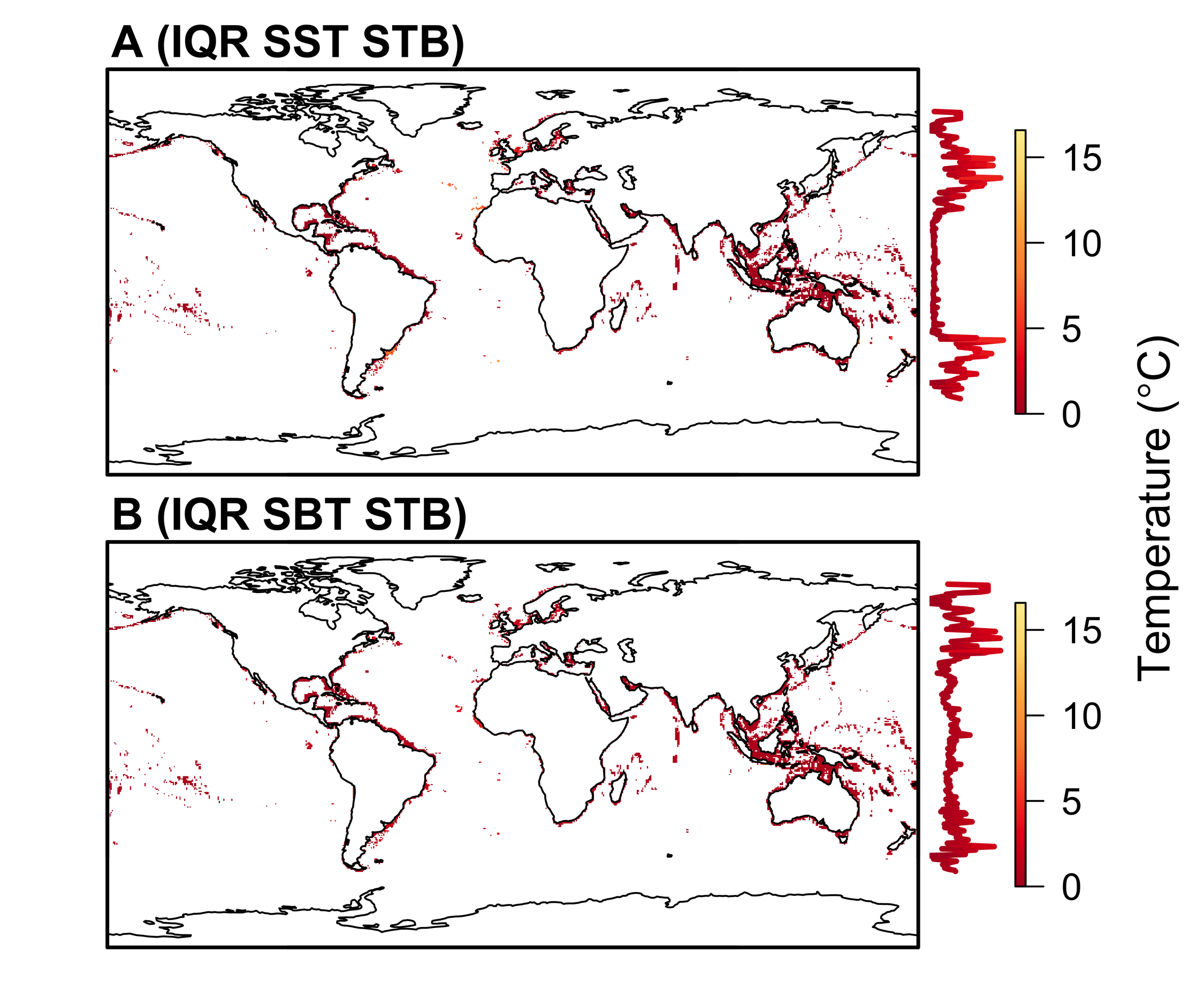

Supplement: S7 Fig — Spatial patterns in the variability of species’ thermal biases (STBs) within Exclusive Economic Zones at 0.5° resolution, derived from baseline (A) sea surface temperature (SST) and (B) sea bottom temperature (SBT) projections. In each grid cell, the interquartile range (IRQ) in thermal bias, calculated over all species whose predicted distributions overlap with that cell, is shown. Adjacent to each map, the coloured line shows the mean IQR across all cells in each latitudinal band, following same colour scheme as for the map. Background coastline data are from Natural Earth (public domain). (TIF) [file pone.0258184.s007.tif]

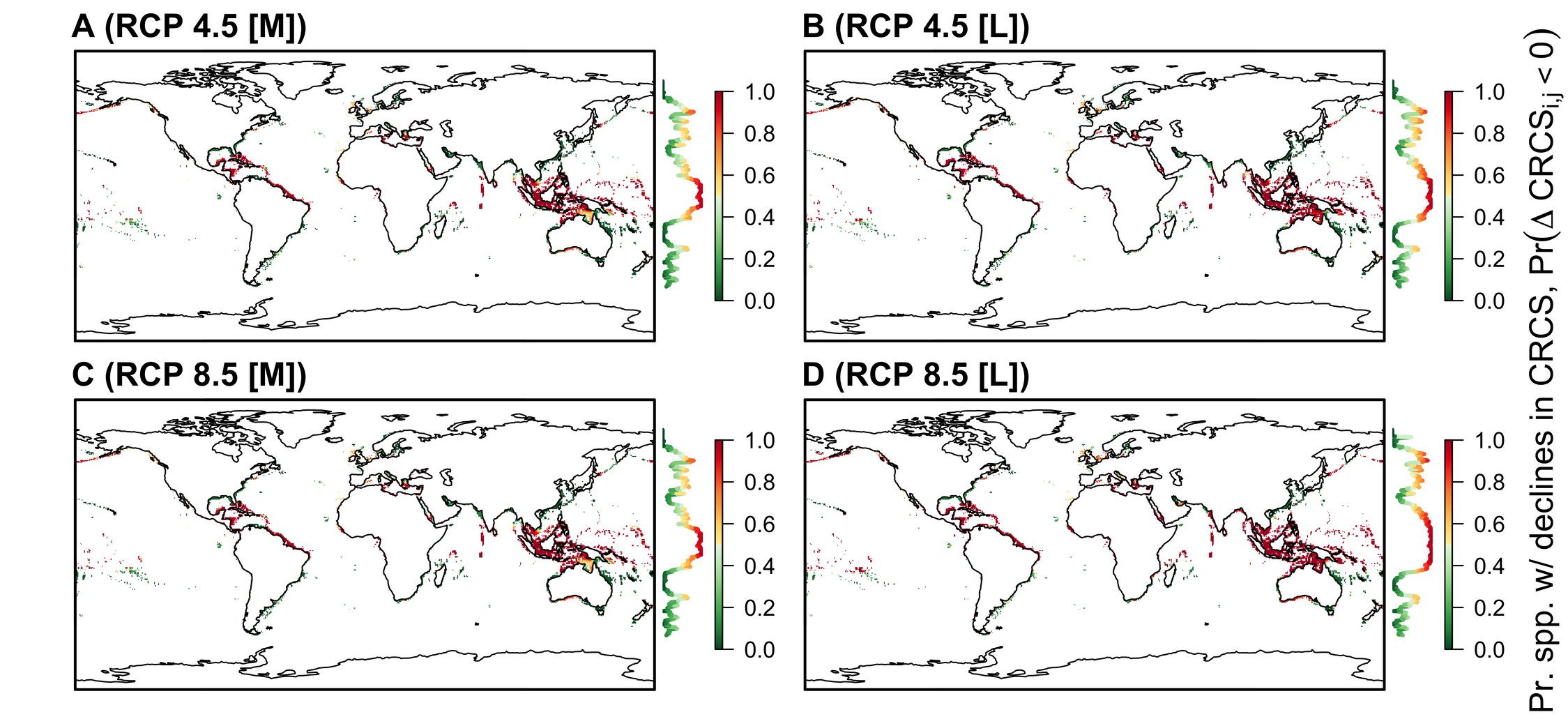

Supplement: S8 Fig — In each 0.5° grid cell, the proportion of species predicted to experience decline in the CRCS, out of the total number of species whose predicted distributions overlap with that cell, is shown. Predictions are only shown within Exclusive Economic Zones. Adjacent to each map, the coloured line shows the mean Pr(ΔCRCSi,j < 0) across all cells in each latitudinal band, following same colour scheme as for the map, with lower proportions (in green) to the left and higher proportions (in red) to the right. Background coastline data are from Natural Earth (public domain). (TIF) [file pone.0258184.s008.tif]

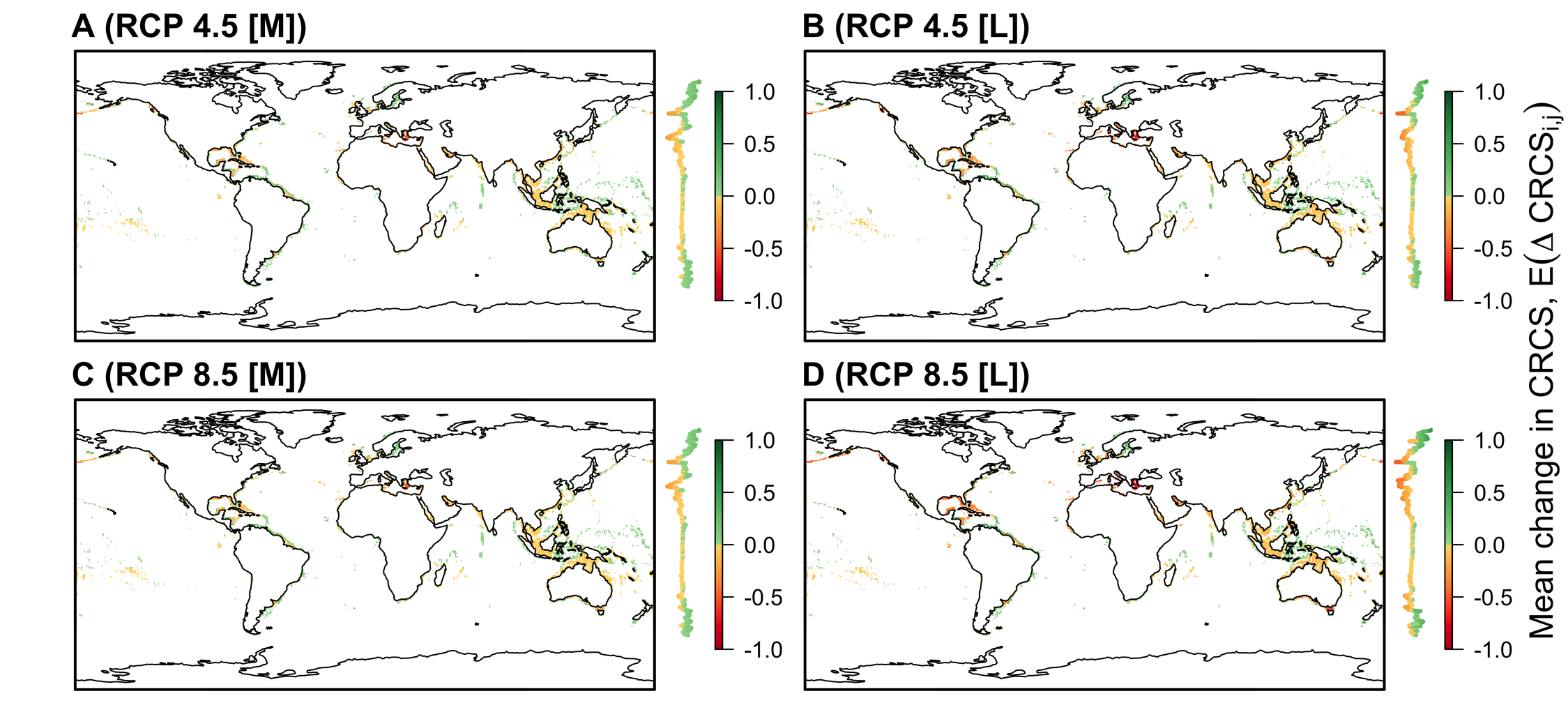

Supplement: S9 Fig — In each 0.5° grid cell, the mean predicted change in the CRCS, calculated across all species whose predicted distributions overlap with that cell, is shown. Predictions are only shown within Exclusive Economic Zones. Adjacent to each map, the coloured line shows the mean E(ΔCRCSi,j) across all cells in each latitudinal band, following same colour scheme as for the map, with lower values (in red) to the left and higher values (in green) to the right. Background coastline data are from Natural Earth (public domain). (TIF) [file pone.0258184.s009.tif]
